# SFTPB in serum extracellular vesicles as a biomarker of progressive pulmonary fibrosis

**DOI:** 10.1172/jci.insight.177937

**Published:** 2024-06-10

**Authors:** Takatoshi Enomoto, Yuya Shirai, Yoshito Takeda, Ryuya Edahiro, Shigeyuki Shichino, Mana Nakayama, Miho Takahashi-Itoh, Yoshimi Noda, Yuichi Adachi, Takahiro Kawasaki, Taro Koba, Yu Futami, Moto Yaga, Yuki Hosono, Hanako Yoshimura, Saori Amiya, Reina Hara, Makoto Yamamoto, Daisuke Nakatsubo, Yasuhiko Suga, Maiko Naito, Kentaro Masuhiro, Haruhiko Hirata, Kota Iwahori, Izumi Nagatomo, Kotaro Miyake, Shohei Koyama, Kiyoharu Fukushima, Takayuki Shiroyama, Yujiro Naito, Shinji Futami, Yayoi Natsume-Kitatani, Satoshi Nojima, Masahiro Yanagawa, Yasushi Shintani, Mari Nogami-Itoh, Kenji Mizuguchi, Jun Adachi, Takeshi Tomonaga, Yoshikazu Inoue, Atsushi Kumanogoh

**Affiliations:** 1 Department of Respiratory Medicine and Clinical Immunology and; 2 Department of Statistical Genetics, Osaka University Graduate School of Medicine, Suita, Osaka, Japan.; 3 Division of Molecular Regulation of Inflammatory and Immune Diseases, Research Institute of Biomedical Sciences, Tokyo University of Science, Chiba, Japan.; 4 Department of Respiratory Medicine, Kinki Central Hospital of the Mutual Aid Association of Public School Teachers, Itami, Hyogo, Japan.; 5 Laboratory of Bioinformatics, Artificial Intelligence Center for Health and Biomedical Research, National Institutes of Biomedical Innovation, Health and Nutrition, Settsu, Osaka, Japan.; 6 Institute of Advanced Medical Sciences, Tokushima University, Tokushima, Japan.; 7 Department of Pathology,; 8 Department of Radiology, and; 9 Department of General Thoracic Surgery, Osaka University Graduate School of Medicine, Suita, Osaka, Japan.; 10 Laboratory for Computational Biology, Institute for Protein Research, Osaka University, Suita, Osaka, Japan.; 11 Laboratory of Proteomics for Drug Discovery, Center for Drug Design Research, National Institutes of Biomedical Innovation, Health and Nutrition, Ibaraki, Osaka, Japan.; 12 Proteobiologics Co., Ltd., Minoh, Osaka, Japan.; 13 Clinical Research Center, NHO Kinki Chuo Chest Medical Center, Sakai, Osaka, Japan.; 14 Osaka Anti-tuberculosis Association, Osaka Fukujuji Hospital, Neyagawa, Osaka, Japan.; 15 Center for Infectious Diseases for Education and Research (CiDER);; 16 Integrated Frontier Research for Medical Science Division, Institute for Open and Transdisciplinary Research Initiatives (OTRI);; 17 Department of Immunopathology, Immunology Frontier Research Center (WPI-IFReC); and; 18 Japan Agency for Medical Research and Development–Core Research for Evolutional Science and Technology (AMED–CREST), Osaka University, Suita, Osaka, Japan.

**Keywords:** Pulmonology, Fibrosis, Proteomics, Pulmonary surfactants

## Abstract

Progressive pulmonary fibrosis (PPF), defined as the worsening of various interstitial lung diseases (ILDs), currently lacks useful biomarkers. To identify novel biomarkers for early detection of patients at risk of PPF, we performed a proteomic analysis of serum extracellular vesicles (EVs). Notably, the identified candidate biomarkers were enriched for lung-derived proteins participating in fibrosis-related pathways. Among them, pulmonary surfactant-associated protein B (SFTPB) in serum EVs could predict ILD progression better than the known biomarkers, serum KL-6 and SP-D, and it was identified as an independent prognostic factor from ILD-gender-age-physiology index. Subsequently, the utility of SFTPB for predicting ILD progression was evaluated further in 2 cohorts using serum EVs and serum, respectively, suggesting that SFTPB in serum EVs but not in serum was helpful. Among SFTPB forms, pro-SFTPB levels were increased in both serum EVs and lungs of patients with PPF compared with those of the control. Consistently, in a mouse model, the levels of pro-SFTPB, primarily originating from alveolar epithelial type 2 cells, were increased similarly in serum EVs and lungs, reflecting pro-fibrotic changes in the lungs, as supported by single-cell RNA sequencing. SFTPB, especially its pro-form, in serum EVs could serve as a biomarker for predicting ILD progression.

## Abstract 2

Nontargeted proteomics of serum extracellular vesicles identifies surfactant-associated protein B, especially its pro-form, as a predictive biomarker for non–idiopathic pulmonary fibrosis interstitial lung diseases.

## Graphical abstract

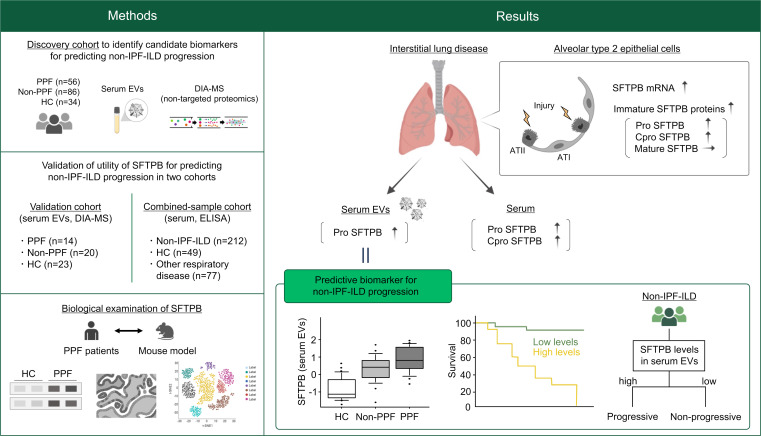

## Introduction

Interstitial lung diseases (ILDs) are a heterogeneous group of parenchymal pulmonary disorders characterized by varying degrees of inflammation and fibrosis (1, 2). Among ILDs, idiopathic pulmonary fibrosis (IPF) shows a typical progressive phenotype, while some patients with non-IPF-ILDs may also exhibit deteriorating lung function and early death (3, 4). Recently, these conditions were termed “progressive pulmonary fibrosis” (PPF) (5), and, similar to IPF, nintedanib was found to be beneficial in the treatment of PPF (6). Currently, PPF can only be diagnosed by monitoring ILD progression based on the past decline in the respiratory function, worsening respiratory symptoms, and disease progression by imaging (5); unfortunately, patients at risk cannot be diagnosed before progression occurs. Nevertheless, for antifibrotic drug effectiveness, early therapeutic intervention is required, emphasizing the value of early PPF diagnosis. However, no reliable biomarker is available for early PPF diagnosis, in other words for predicting the degree of non-IPF-ILD progression.

Based on recent progress in next-generation sequencing and mass spectrometry (MS), increased attention is being paid to omics and biomarker discovery to determine personalized medicine for patients with various disorders (7). Peripheral blood has been recognized as an ideal source of biomarkers because of its accessibility and measurement reproducibility. However, approximately 22 proteins, including albumin, immunoglobulins, and complement factors, comprise 99% of the total proteins, thereby masking the small numbers of other biomarker candidates when measured by conventional proteomics (8).

Extracellular vesicles (EVs) are nanometer-sized vesicles secreted by most cell types that contain several biomolecules, contributing to various physiological functions (9). Contrary to the serum, their cargo is protected from protease and nuclease degradation by lipid bilayers, and EVs are free of foreign substances, facilitating the detection of proteins in trace amounts by proteomics (8, 9). Thus, EVs could be regarded as ideal sources of biomarkers. Notably, previous studies of EVs have identified crucial biomarkers in various diseases, including ILDs (10–13); however, there is a lack of studies using EVs to identify biomarkers of PPF.

Thus, we sought to identify specific PPF biomarkers that could predict the degree of non-IPF-ILD progression. First, we performed a proteomic analysis of serum EVs and evaluated the clinical features of candidate biomarkers in a discovery cohort. Next, we tested the reproducibility of the clinical utility of the thus-identified biomarker in the validation and combined-sample cohorts, using serum EVs and serum, respectively. Finally, we examined the temporal dynamics and functions of the identified biomarker during the development of pulmonary fibrosis using samples from patients with PPF and a bleomycin-induced pulmonary fibrosis mouse model. A flowchart of this study is shown in Supplemental Figure 1; supplemental material available online with this article; https://doi.org/10.1172/jci.insight.177937DS1

## Results

### Participant demographics.

The clinical characteristics at the time of blood collection of the discovery and validation cohorts are shown in Table 1 and Supplemental Table 1, respectively. In the discovery cohort, compared with the non-PPF group, the PPF group had patients who were younger, had a greater proportion using antifibrotic drugs, had a higher proportion with idiopathic nonspecific interstitial pneumonia (INSIP) and fibrosing hypersensitivity pneumonitis (FHP), had a lower proportion with unclassifiable ILD, and showed a greater extent of fibrosis on the CT scan. In the validation cohort, compared with the non-PPF group, the PPF group had patients who were younger, had a lower proportion with collagen vascular disease–associated ILD (CVD-ILD), and showed a greater extent of fibrosis on the CT scan.

### Screening of PPF biomarkers in the discovery cohort.

**Figure 1:**
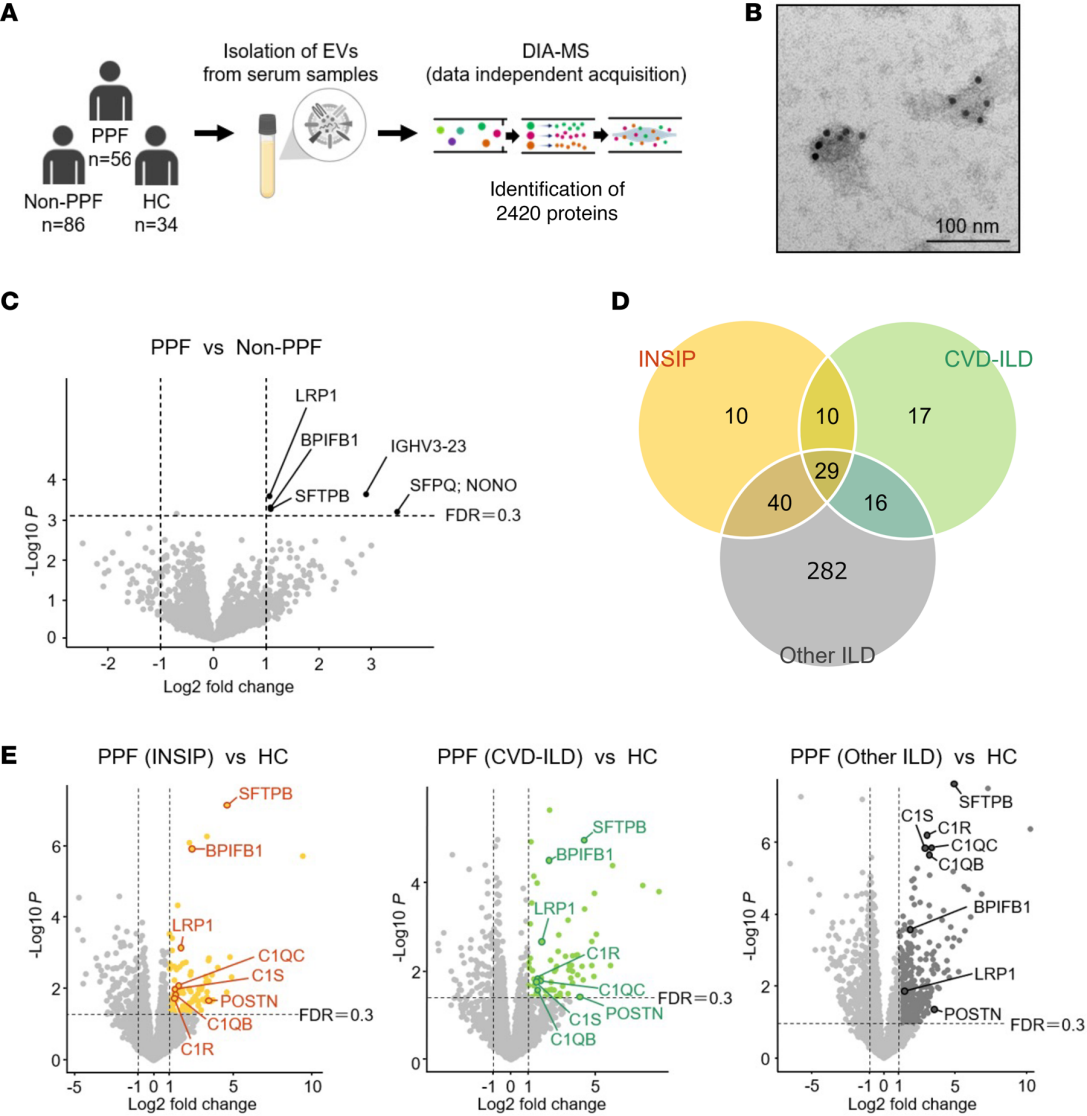
Screening of PPF biomarkers in the discovery cohort. (**A**) Overview of the project design in the discovery cohort. Serum extracellular vesicles (EVs) from 56 progressive pulmonary fibrosis (PPF) cases, 86 non-PPF cases, and 34 healthy controls (HCs) were analyzed by data-independent acquisition (DIA). (**B**) Representative transmission electron microscopy images of serum EVs from an HC: Immunogold labeling (BBI International) with CD9. Scale bar, 100 nm. (**C**) Identification of differentially expressed proteins in serum EVs in PPF as compared with those in non-PPF. (**D** and **E**) Identification of proteins in serum EVs, whose expression was increased in 3 PPF groups — idiopathic nonspecific interstitial pneumonia (INSIP) (*n* = 22), collagen vascular disease–associated interstitial lung disease (CVD-ILD) (*n* = 14), and other ILD (*n* = 20) — as compared with that in HCs (*n* = 34).

First, we performed a proteomic analysis of serum EVs in the discovery cohort. We identified 2,420 proteins in serum-derived EVs from less than 20 μL of serum from 56 PPF cases, 86 non-PPF cases, and 34 healthy controls (HCs) using liquid chromatography with tandem mass spectrometry (LC-MS/MS) analysis (Figure 1A). The serum EVs isolated from the PPF cases, non-PPF cases, and HCs expressed the general EV marker CD9 on their surfaces (Figure 1B). Additionally, the isolated EVs were positive for CD9 and Flotillin-1 and negative for Calnexin and Apolipoprotein A1 (Supplemental Figure 2A). They were also of comparable size and abundance (Supplemental Figure 2, B–F).

Next, we evaluated the protein variation between the PPF and the non-PPF groups to identify ILD progression-related biomarkers. Consequently, the levels of 5 proteins, such as pulmonary surfactant-associated protein B (SFTPB; also known as surfactant protein B) (log_2_ fold-change [FC] 1.06; –log_10_*P* 3.62) and pro low-density lipoprotein receptor-related protein 1 (LRP1) (log_2_ FC 1.08; –log_10_*P* 3.30), were significantly increased in the PPF compared with the non-PPF group (Supplemental Table 2 and Figure 1C). We further evaluated the protein variation between the PPF and HC group. To search for biomarkers related to common progressive fibrosis in PPF with high heterogeneity, PPF cases were divided into 3 representative groups — INSIP, CVD-ILD, and other ILD — and protein variation, which was common to the 3 groups as compared with the HC group, was evaluated. In the INSIP, CVD-ILD, and other ILD groups, the levels of 89, 72, and 367 proteins were significantly increased, respectively (Figure 1, D and E). Notably, the levels of 29 proteins, including SFTPB, were increased in all 3 groups (Supplemental Table 3). Meanwhile, in the INSIP, CVD-ILD, and other ILD groups, the levels of 111, 79, and 262 proteins were decreased, respectively, compared with those in the HC group (Supplemental Figure 3, A and B). The levels of 30 proteins were commonly decreased in these groups (Supplemental Table 4).

### Protein signature in serum EVs reflecting the PPF pathogenesis.

**Figure 2:**
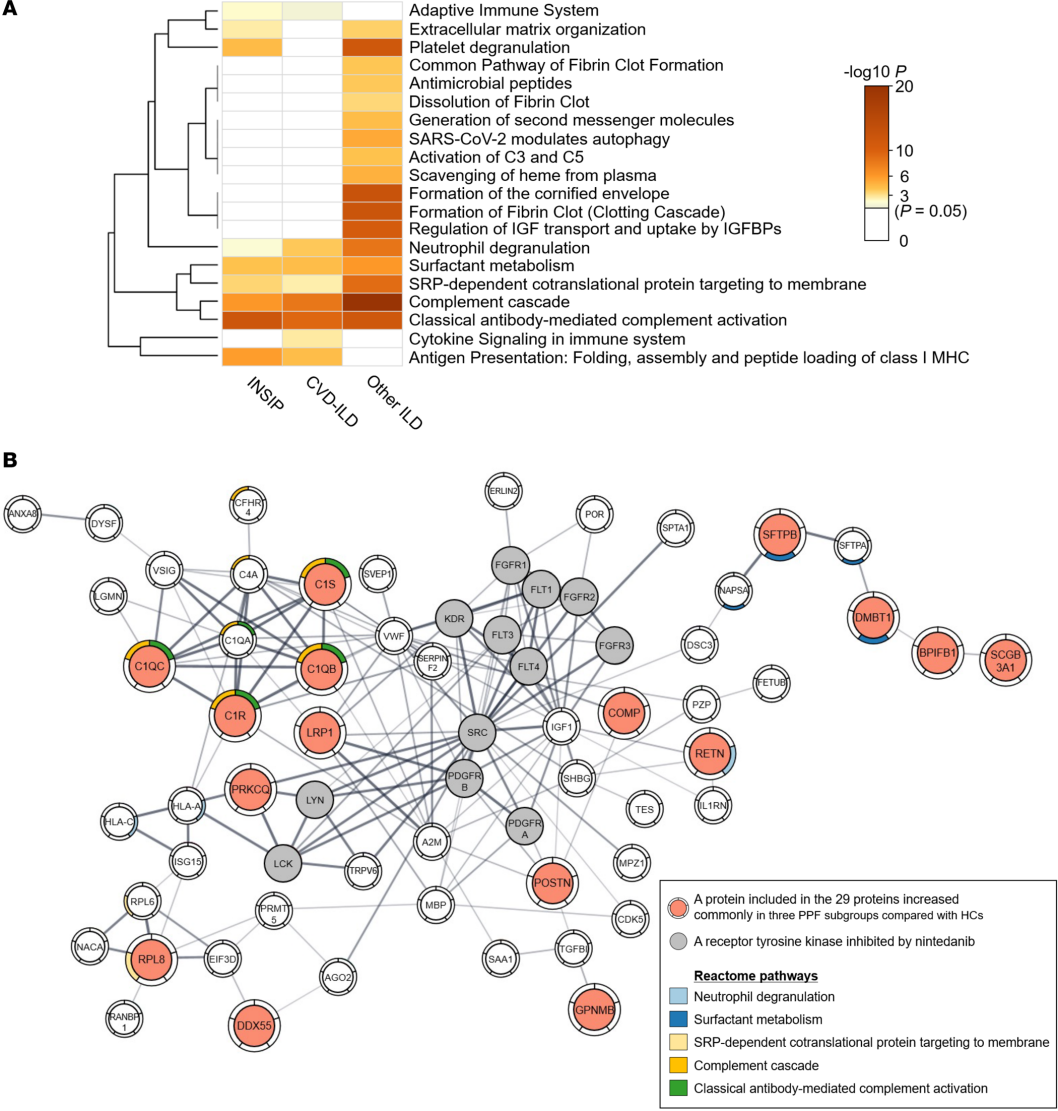
Proteomic profiling of candidate biomarkers in the discovery cohort. (**A**) Reactome pathway enrichment analysis of the proteins, the levels of which were increased in 3 PPF subgroups compared with HCs. These proteins were commonly enriched in pulmonary fibrosis–related pathways, such as neutrophil degranulation and surfactant metabolism. (**B**) Protein–protein interaction analysis using the STRING database among the 95 biomarker candidates whose expression was commonly increased in at least 2 PPF groups compared with those in HCs, together with tyrosine kinases inhibited by nintedanib. Here, only proteins that interact with tyrosine kinases inhibited by nintedanib are depicted.

By reactome pathway enrichment analysis using Metascape (14), the increased proteins in the INSIP, CVD-ILD, and other ILD groups compared with the HC group were found to be commonly enriched for fibrosis-related pathways, such as neutrophil degranulation and surfactant metabolism (Figure 2A). To estimate the potential origin of the 95 biomarker candidates whose expression increased commonly in at least 2 groups of PPF compared with those in the HC group, we analyzed their mRNA expression based on RNA consensus tissue gene data from the Human Protein Atlas (Supplemental Figure 4A). We observed that these candidate biomarkers were enriched for lung-specific proteins compared with the 2,420 identified proteins (Supplemental Figure 4B). To further examine the associations between the candidate biomarkers and PPF pathogenesis, we analyzed the protein–protein interactions between the 95 identified biomarker candidates and tyrosine kinases inhibited by nintedanib (15). Notably, we identified close interactions between them. These results suggested that the candidate biomarkers in serum EVs could be involved in the PPF pathogenesis (Figure 2B).

### Clinical features of SFTPB in serum EVs.

**Figure 3:**
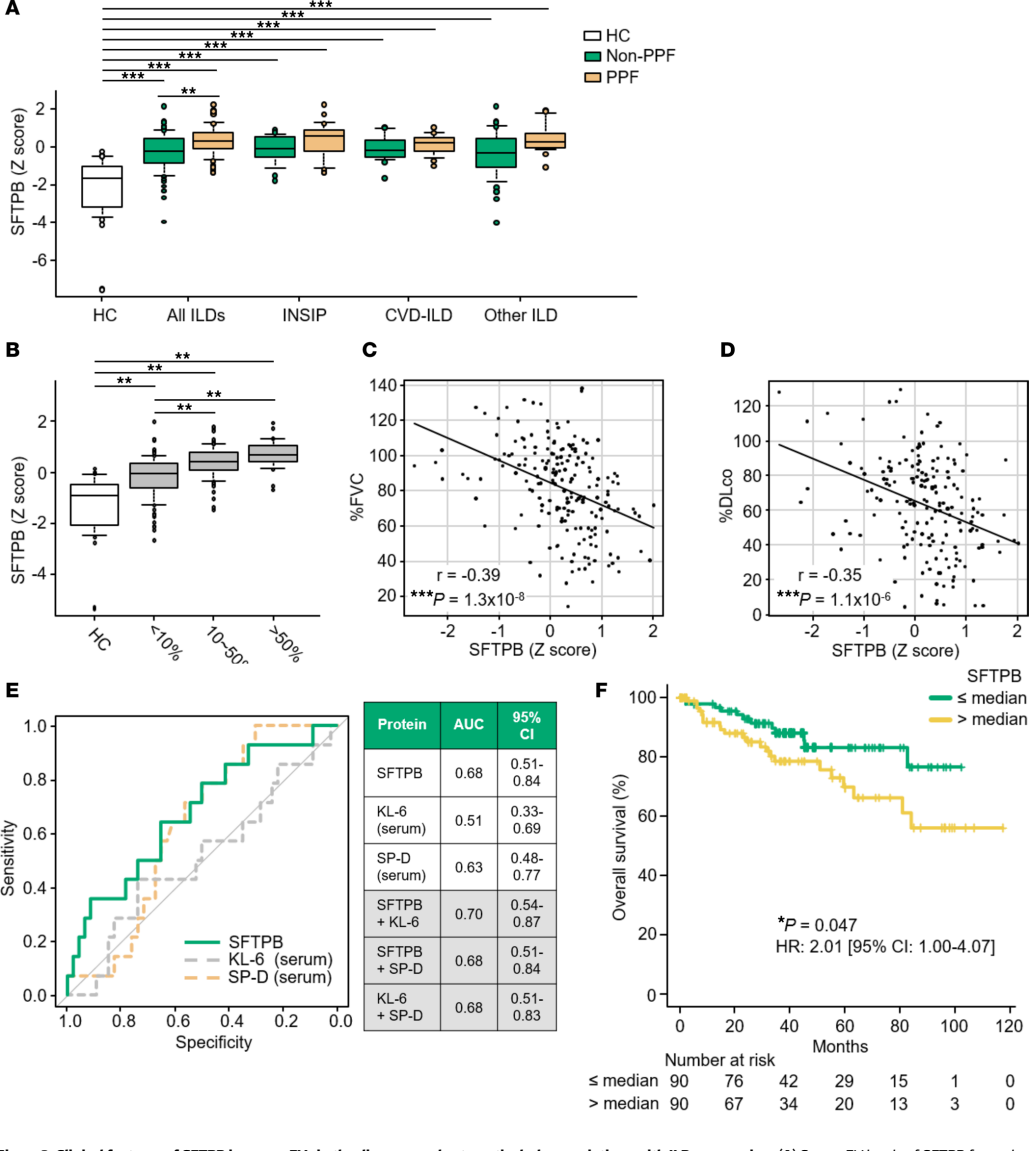
Clinical features of SFTPB in serum EVs in the discovery cohort, particularly associations with ILD progression. (**A**) Serum EV levels of SFTPB for each disease, measured by DIA in the discovery cohort. Pairwise intergroup comparisons between HCs and each ILD subgroup, as well as between non-progressive pulmonary fibrosis (non-PPF) and PPF in all ILDs, INSIP, CVD-ILD, and other ILD groups were performed using 2-tailed Student’s *t* test (Bonferroni correction), with **P < 0.01, and ***P < 0.001. (**B**) SFTPB levels in serum EVs correlated with the extent of interstitial shadows on CT scans. The differences were analyzed by ANOVA, and Holm’s method was applied to adjust for *P* values: **P < 0.01. (**A** and **B**) The boxes indicate interquartile ranges (75% and 25%) and medians; the upper and lower whiskers represent the 10% and 90% points, respectively. (**C** and **D**) Pearson correlations of SFTPB with percentage predicted forced vital capacity (%FVC) and the diffusing capacity for carbon monoxide (%DLco). ***P < 0.001. (**E**) Receiver operating characteristic (ROC) curves for evaluating SFTPB in serum EVs, serum KL-6, and SP-D as predicting composite outcome (relative decline in %FVC ≥ 10%, acute exacerbation, or death) within a year in 60 evaluable ILD cases in the discovery cohort. (**F**) Kaplan-Meier curve estimating the probability of overall survival (OS) stratified by the SFTPB levels in serum EVs. In 180 cases with INSIP, CVD-ILD, FHP, or unclassifiable ILD, high levels of SFTPB in serum EVs were significantly associated with high mortality. OS was defined as the period from the date of blood collection to the date of death from any cause.

Among the identified candidate biomarkers, the levels of SFTPB were increased in the non-PPF group versus in HCs and trended to be further increased in PPF (Figure 3A, Supplemental Table 5). The clinical characteristics for each ILD classification are shown in Supplemental Table 6. We then added 78 non-IPF-ILD cases that could not be evaluated for PPF or non-PPF because of missing data of the patients to assess the clinical features of SFTPB. The levels of SFTPB tended to increase with the extent of interstitial shadows (Figure 3B, Supplemental Table 7). Consistently, SFTPB levels showed negative correlations with percentage predicted forced vital capacity (%FVC) and percentage diffusing capacity for carbon monoxide (%DLco) (Figure 3, C and D).

### SFTPB in serum EVs as a biomarker for the prediction of non-IPF-ILD progression.

We subsequently sought to examine the prognostic potential of SFTPB in serum EVs. Here, to reduce the prognostic impact of ILD heterogeneity (16), we limited our analysis to the patients with ILD including INSIP, CVD-ILD, FHP, and unclassifiable ILD (16). According to univariate analysis using a logistic regression model, among clinical components (age, sex, smoking history, %FVC, and %DLco), %FVC was significantly associated with future ILD progression within 1 year (odds ratio: 0.96, *P* = 0.0074). Notably, among the biomarkers, only SFTPB was significantly associated with future ILD progression (odds ratio: 1.69, *P* = 0.042), though in multivariate analysis with %FVC, it was not (odds ratio: 1.41, *P* = 0.20) (Supplemental Table 8). Consistently, according to receiver operating characteristic (ROC) curve analysis, SFTPB in serum EVs was superior to serum KL-6 and SP-D in predicting future ILD progression within 1 year (AUC: 0.68 versus 0.51 and 0.63, respectively). Among combinations of any 2 biomarkers, combining SFTPB and KL-6 resulted in the best performance (AUC: 0.70) (Figure 3E). In addition, in 180 patients with ILD, high levels of SFTPB in serum EVs were significantly associated with mortality (hazard ratio [HR] 2.01; 95% confidence interval [95%CI] 1.00–4.07; *P* = 0.047 by log-rank test) (Figure 3F). In 158 patients with complete data for all variables, univariate analysis using a Cox proportional hazard regression model indicated that SFTPB level in serum EVs, %DLco, and ILD-gender-age-physiology (ILD-GAP) index were significantly associated with mortality (Table 2). Furthermore, multivariate analysis using stratified Cox proportional hazard regression model by ILD-GAP index identified SFTPB level in serum EVs as an independent prognostic factor from smoking history and ILD-GAP index (HR 2.65; 95%CI 1.04–6.74; *P* = 0.041) (Table 2). In subgroup analyses by ILD-GAP index, high levels of SFTPB in serum EVs tended to be associated with high mortality in ILD-GAP index 0–1 and 2–3 groups but not 4–5 group (Supplemental Figure 5, A–C). In summary, SFTPB in serum EVs could be helpful for predicting ILD progression in non-IPF-ILD.

### Validation of associations of SFTPB levels in serum EVs with non-IPF-ILD progression in the validation cohort.

**Figure 4:**
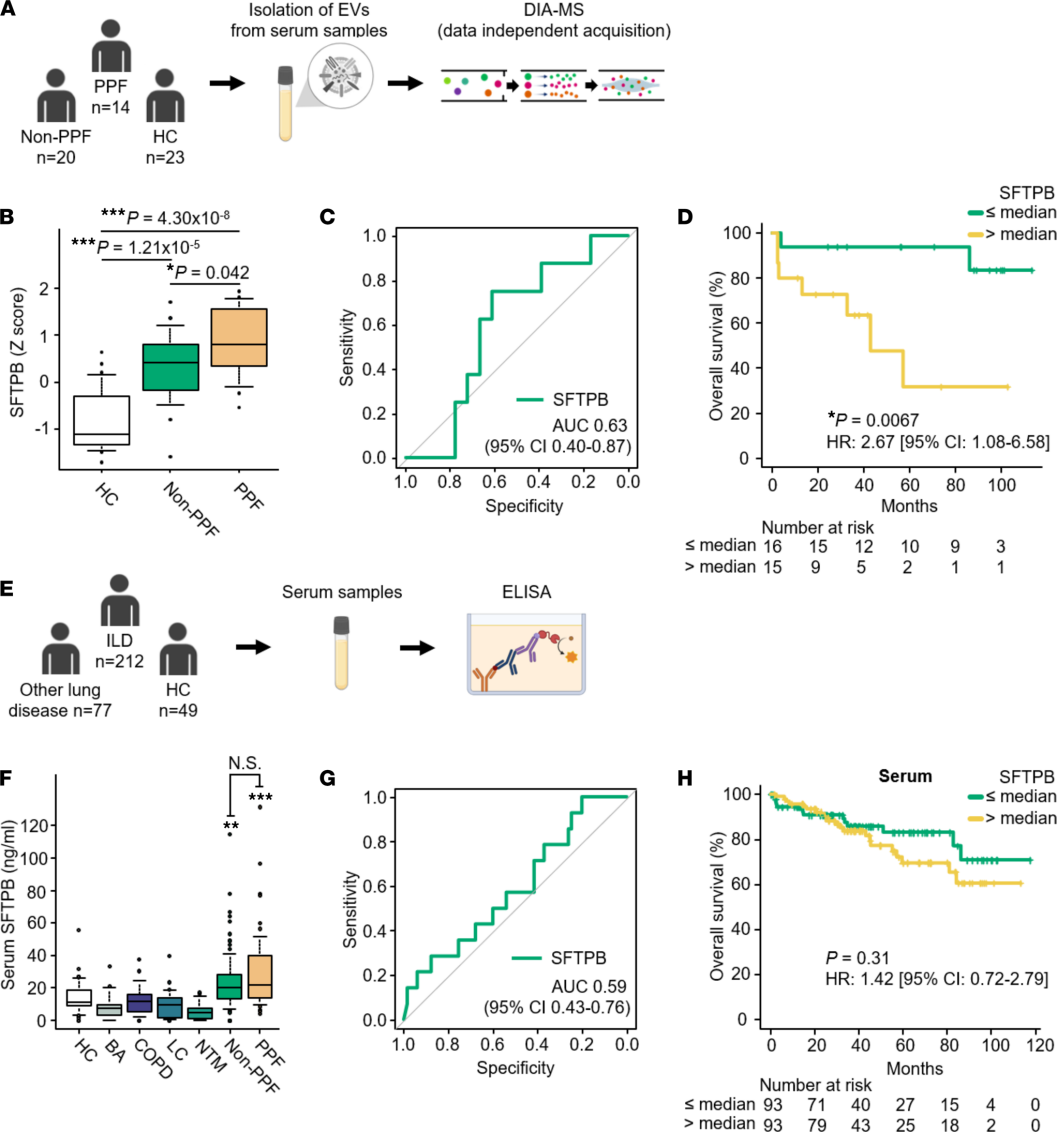
Reproducibility of associations between SFTPB in serum EVs but not in serum and ILD progression. (**A** and **E**) Schematic representation of the project designs in the validation cohort (**A**) and in the combined-sample cohort (**E**). In the validation cohort, serum EV levels of SFTPB were analyzed by data-independent acquisition. In the combined-sample cohort, serum levels of SFTPB were measured by ELISA. (**B**) Serum EV levels of SFTPB in the validation cohort. Numbers of samples: HC (*n* = 23), non-PPF (*n* = 20), PPF (*n* = 14). The data were subjected to ANOVA, and Holm’s method was applied to adjust for the ANOVA *P* values. (**F**) Serum levels of SFTPB in the combined-sample cohort. Numbers of samples: HC (*n* = 49), BA (*n* = 20), COPD (*n* = 20), LC (*n* = 22), NTM (*n* = 15), non-PPF (*n* = 100), and PPF (*n* = 60). The expression levels were compared by ANOVA, and Dunnett’s method was applied to adjust for the ANOVA *P* values. Subsequently, differences between PPF and non-PPF were compared by ANOVA, and Holm’s method was applied to adjust for the ANOVA *P* values. (**B** and **F**) *P < 0.05, **P < 0.01, and ***P < 0.001 for significant differences from healthy control. N.S., no significant difference between non-PPF and PPF. (**C** and **G**) ROC curves for evaluating SFTPB in serum EVs as predicting composite outcome (relative decline in %FVC ≥ 10%, acute exacerbation, or death) within a year in 23 evaluable ILD cases in the validation cohort (**C**) and ROC curves for evaluating SFTPB in serum as predicting composite outcome in 78 evaluable ILD cases in the combined-sample cohort (**G**). (**D** and **H**) Kaplan-Meier curves estimating the probability of overall survival (OS) stratified by the serum EV levels of SFTPB in the validation cohort (**D**) and the serum levels of SFTPB in the combined-sample cohort (**H**). OS was defined as the period from the date of blood collection to the date of death from any cause. BA, bronchial asthma; COPD, chronic obstructive pulmonary disease; LC, lung cancer; NTM, nontuberculous mycobacterial lung disease.

We further measured SFTPB levels in serum EVs from 14 PPF cases, 20 non-PPF cases, and 23 HCs of the validation cohort using LC-MS/MS analysis (Figure 4A). Consistently, SFTPB levels in serum EVs were significantly increased in the non-PPF group compared with those in the HC group and further increased in the PPF group (Figure 4B). According to ROC analysis for predicting ILD progression, the AUC value of SFTPB obtained with the discovery cohort was generally reproduced (AUC: 0.63) (Figure 4C). Furthermore, high levels of SFTPB in serum EVs were significantly associated with mortality (HR 2.67; 95%CI 1.08–6.58; *P* = 0.0067 by log-rank test) (Figure 4D), and stratified Cox proportional hazards analysis by ILD-GAP index indicated a similar trend (HR 4.37; 95%CI 0.89–21.35; *P* = 0.069). Subgroup analyses by ILD-GAP index indicated that in ILD-GAP index 0–1 and 2–3 groups, high levels of SFTPB in serum EVs tended to be associated with high mortality (Supplemental Figure 6, A and B).

### Validation of associations of SFTPB levels in serum measured by ELISA with non-IPF-ILD progression in the combined-sample cohort.

Given that some biomarker candidates identified in EVs could also be detected in the serum (12), we next sought to measure serum levels of SFTPB by ELISA (Figure 4E). The serum levels of SFTPB were specifically increased in ILD cases but not in other respiratory diseases, such as bronchial asthma (BA), chronic obstructive pulmonary disease (COPD), and lung cancer. However, the serum levels in the PPF group were not significantly increased compared with the non-PPF group (Figure 4F). According to ROC analysis for predicting ILD progression, the AUC value of SFTPB levels in serum was inferior to those in serum EVs obtained with the discovery and validation cohorts (Figure 4G). Furthermore, high levels of serum SFTPB were not significantly associated with mortality (HR 1.42; 95%CI 0.72–2.79; *P* = 0.31) (Figure 4H). These results further emphasize the superiority of analyzing SFTPB levels in serum EVs to that in the serum for predicting non-IPF-ILD progression.

### Localization of SFTPB in lung tissues of patients with PPF.

**Figure 5:**
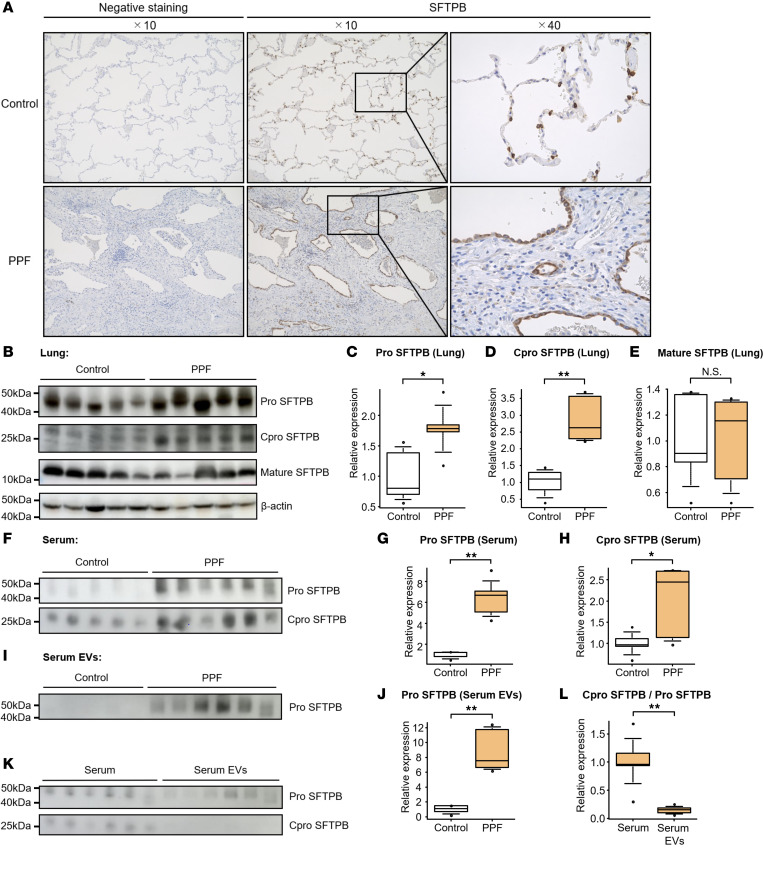
Increased levels of immature SFTPB protein in patients with PPF. (**A**) A representative image of immunohistochemistry for SFTPB using lung sections from controls and PPF cases. In controls, SFTPB-positive alveolar epithelial cells were scattered over the alveolar surface, while SFTPB-positive alveolar epithelial cells with reactive atypia covered the alveolar surface in fibrotic areas. (**B**–**E**) Western blotting for evaluating SFTPB in 10 lung tissue specimens, including 5 PPF surgical specimens and 5 control tissues (surgical specimens from patients with lung cancer). (**F**–**H**) Western blotting for evaluating SFTPB in the serum of HCs (*n* = 5) and PPF cases (*n* = 6). (**I** and **J**) Western blotting for evaluating SFTPB in serum EVs from HCs (*n* = 5) and PPF cases (*n* = 6). The same samples were used as in the study of serum. (**K** and **L**) Western blotting for evaluating SFTPB between the serum and serum EVs in PPF cases (*n* = 6). The same samples were used as in the studies of serum and serum EVs. Serum samples per lane were generated from 0.01 μL of serum, while serum EV samples per lane were generated from 70 μL of serum. (**B**–**L**) Patient characteristics are shown in Supplemental Tables 9 and 10. The intensity of the SFTPB band was evaluated using ImageJ (NIH) and normalized to levels of β-actin in lung tissue specimens. Differences between 2 groups were compared using 2-tailed Student’s *t* test. *P < 0.05; **P < 0.01.

By immunohistochemistry, SFTPB expression was increased in alveolar and airway epithelial cells. In particular, we found SFTPB-positive epithelial cells with reactive atypia covered the alveolar surface in the fibrotic area (Figure 5A). In a single-cell RNA-sequencing (scRNA-Seq) data set (17), *SFTPB* was mainly expressed in alveolar epithelial type 2 (ATII) cells, alveolar type 1 epithelial cells, and club cells (Supplemental Figure 7, A–C), consistent with our immunohistochemistry findings.

### Increased levels of immature SFTPB in patients with PPF.

Considering that SFTPB acquires several forms during its complex multistep maturation process and that proteomics cannot discriminate the differences in SFTPB forms (18), we further performed Western blotting. In PPF lungs, the levels of pro-SFTPB and Cpro-SFTPB, i.e., immature SFTPB, increased, while those of mature SFTPB did not (Figure 5, B–E). In the serum, pro- and Cpro-SFTPB levels increased in PPF similar to that in lung tissues, but mature SFTPB was not detected (Figure 5, F–H). In serum EVs, while pro-SFTPB levels were increased in PPF similar to those in lung tissues and serum (Figure 5, I and J), mature SFTPB was not detected; notably, Cpro-SFTPB was also barely detectable, unlike that in the serum (Figure 5, K and L). Thus, immature SFTPB levels were commonly increased in lungs, serum, and serum EVs. Particularly, the increase in SFTPB levels in serum EVs could specifically represent the increase in the SFTPB pro-form among various forms of SFTPB.

### Pro-SFTPB levels in serum EVs are positively associated with those in lung tissues during progressive fibrosis in a mouse model.

**Figure 6:**
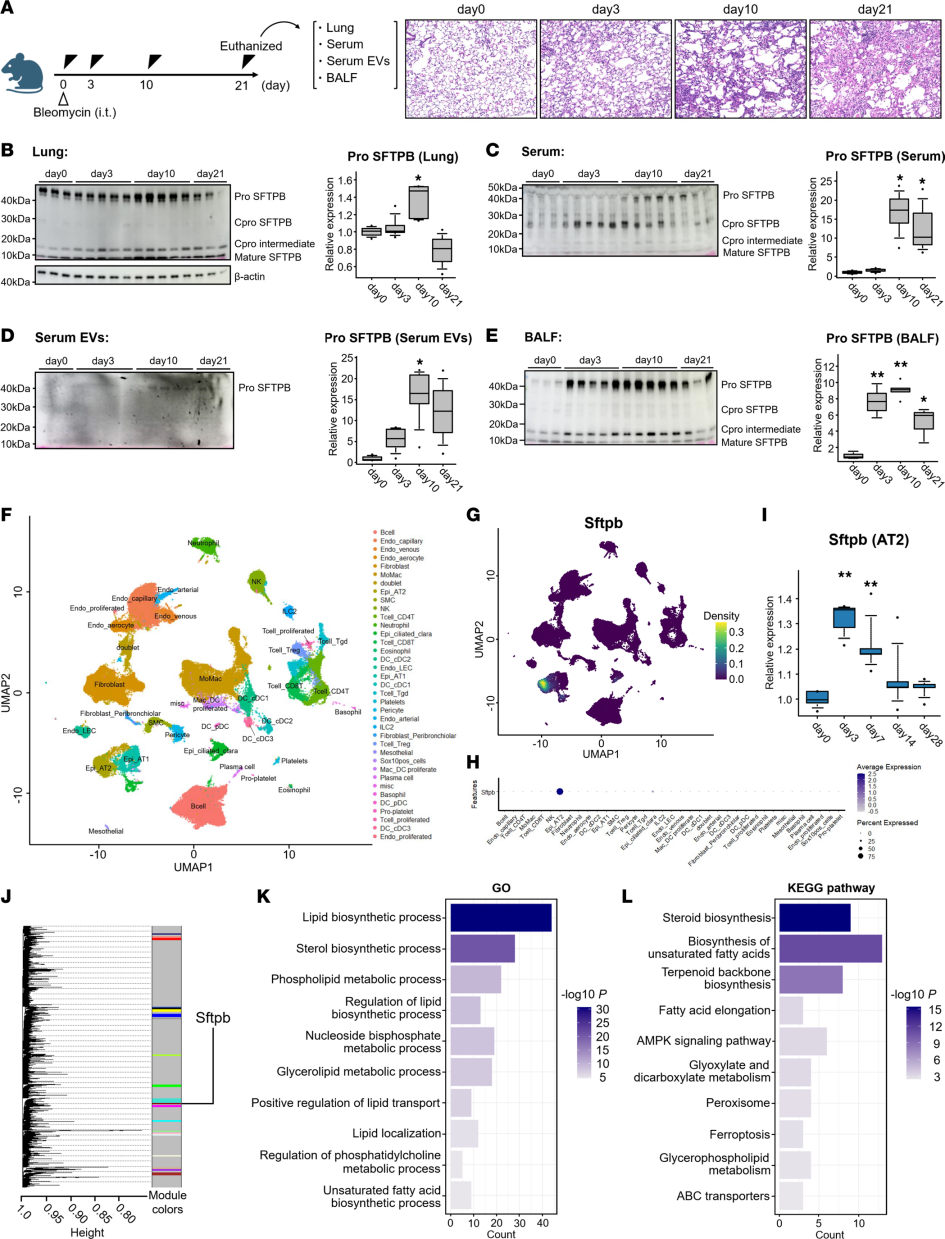
Spatial and longitudinal dynamics of SFTPB during the development of bleomycin-induced lung fibrosis. (**A**) Schematic representation of the experimental protocol used for Western blotting analysis of lung tissues, serum, serum EVs, and BALF from bleomycin-induced pulmonary fibrosis model mice and representative hematoxylin-eosin staining of lung tissues. (**B**–**E**) Western blotting analysis of SFTPB in lung tissues, serum, serum EVs, and BALF and quantification of the levels of pro-SFTPB. *n* = 3–5 mice per group. The level of pro-SFTPB was increased, peaking on day 10, across all source materials. (**F**) Uniform manifold approximation and projection (UMAP) embedding of single-cell transcriptomes from 77,656 cells from 5 control mice (on day 0) and 20 bleomycin-induced mouse lungs (on days 3, 7, 14, 28, *n* = 5 mice per group) annotated by cell type. (**G** and **H**) Density and dot plots of *Sftpb* mRNA expression levels. (**I**) Changes in the expression of *Sftpb* mRNA in alveolar type 2 epithelial cells (AT2) by pseudo-bulk analysis. *n* = 5 mice per group. (**J**) Dendrogram of high dimensional weighted correlation network analysis in AT2 cells of mice on days 0, 3, and 7. (**K** and **L**) The 10 most significantly (*P* < 0.05) enriched terms in GO biological process and KEGG pathways in 126 genes covarying with *Sftpb*. (**B**–**E** and **I**) The boxes indicate interquartile ranges (75% and 25%) and medians; the upper and lower whiskers represent the 10% and 90% points, respectively. The expression levels were compared by ANOVA, and Dunnett’s method was applied to adjust for the ANOVA *P* values. *P < 0.05; **P < 0.01.

To evaluate the longitudinal expression of SFTPB in lung, serum, serum EVs, and bronchoalveolar lavage fluid (BALF) during the development of fibrosis, we utilized a mouse model of bleomycin-induced pulmonary fibrosis (Figure 6A). Consistent with those in patients with PPF, pro-SFTPB levels were increased in lung tissues, peaking on day 10 (Figure 6B). A similar behavior of pro-SFTPB was observed in the serum, serum EVs, and BALF (Figure 6, C–E). Meanwhile, several other SFTPB forms exhibited more complex dynamics. For example, Cpro-SFTPB responded more quickly in the serum, and its levels were increased, peaking on day 3 (Figure 6C), whereas in BALF, mature SFTPB levels were not increased after bleomycin administration (Figure 6E). Consistent with the results in human samples, only the pro-form was detected for SFTPB in serum EVs.

### Upregulation of Sftpb mRNA expression is associated with fibrosis-related pathways.

To obtain mechanistic insights regarding the function of SFTPB in fibrotic lung tissues, we performed scRNA-Seq. *Sftpb* mRNA was expressed mainly in ATII cells (Figure 6, F–H). Pseudo-bulk analysis revealed that *Sftpb* expression increased in ATII cells on days 3 and 7 after bleomycin administration (Figure 6I). Although *Sftpd* was upregulated, peaking on day 3, *Sftpa1* and *Sftpc* expression transiently decreased (Supplemental Figure 8, A–C). To estimate the function of upregulated *Sftpb* in ATII cells, we performed high dimensional weighted gene correlation network analysis (hdWGCNA) in mouse ATII cells on days 0, 3, and 7 (19). Consequently, we detected that the expression of a set of 126 genes covaried with the expression of *Sftpb* (Figure 6J). Both Gene Ontology (GO; biological process) and Kyoto Encyclopedia of Genes and Genomes (KEGG) pathway enrichment analyses of these covarying genes revealed enrichment in lipid-related terms, such as lipid biosynthetic process and biosynthesis of unsaturated fatty acids. Furthermore, KEGG pathway enrichment analysis revealed associations with pathways involved in pulmonary fibrosis, such as AMPK signaling pathway, along with lipid-related pathways (20–22). Taken together, these results indicate that *Sftpb* could be involved in pulmonary fibrosis through these pathways (Figure 6, K and L).

## Discussion

Herein, we performed non-targeted proteomics of serum EVs to identify novel PPF biomarkers. We found that serum EVs constitute a source of protein biomarkers related to the fibrogenic pathogenesis of PPF. Notably, SFTPB in serum EVs was identified as a biomarker for predicting non-IPF-ILD progression and an independent prognostic factor from ILD-GAP index. SFTPB in serum EVs has the potential to enable stratification of people at risk for non-IPF-ILD progression, leading to early and appropriate therapeutic intervention.

In this study, we used the DIA method of serum EVs and managed to identify and quantify more than 2,000 proteins, exceeding the number of proteins identified in previous studies (23, 24). Recently, a study reported biomarkers predicting survival in IPF using a high-throughput proteomics platform, Olink, which could quantify approximately 3,000 proteins (25); however, Olink is a targeted proteomics approach, which measures the levels of proteins in the defined panels. SFTPB is not currently included in the default panels and, therefore, was not measured in this prior study. Our bias-free biomarker search using a non-targeted proteomics approach by DIA contributed to the identification of SFTPB as a predictive biomarker of ILD progression.

Proteomic analysis of serum EVs showed that the identified biomarker candidates in PPF were enriched in lung-specific proteins. In addition, those proteins were associated with pulmonary fibrosis–related pathways, such as neutrophil degranulation and surfactant metabolism (26), and interacted with tyrosine kinases inhibited by nintedanib (15). These results support that EVs could be used as liquid biopsies for understanding the pathogenesis of lung fibrosis.

SFTPB is a member of the protein families SP-A, -B, -C, and -D, which, together with phospholipids, constitute surfactants (27). SFTPB contributes to surface tension reduction at the lung air–liquid interface; newborn babies bearing *SFTPB* mutations develop respiratory distress syndrome (28). SFTPB undergoes a complex multistep maturation process, which occurs both intra- and extracellularly (18), producing the SFTPB precursor and mature proteins, together with their processing intermediates, in the alveolar epithelial lining fluid (29). While the mature SFTPB is too hydrophobic to circulate in the bloodstream, immature SFTPB proteins are less hydrophobic, and are detectable in the serum, where they might serve as biomarkers for pulmonary diseases with alveolar or interstitial damage, such as ILDs and especially IPF (30, 31). However, in contrast with SP-A and D, reports of SFTPB as an ILD-related biomarker remain limited (32). In the present study, SFTPB in serum EVs was identified as an independent prognostic factor from ILD-GAP index, rather than merely exhibiting a correlation with disease severity. Although SFTPB in serum EVs did not demonstrate clinically adequate performance to conclusively predict ILD progression, it exhibited superiority over the known biomarkers, serum KL-6 and SP-D. To the best of our knowledge, our study is the first to report SFTPB in serum EVs as a predictive biomarker of non-IPF-ILD progression. Furthermore, our study revealed that while pro- and Cpro-SFTPB levels were increased in PPF serum, pro-SFTPB levels were increased in serum EVs, whereas other forms were barely detected. These results suggest the following possibilities. First, when SFTPB is released wrapped in EVs, it may exist predominantly in its pro-form. Second, the SFTPB within EVs may be protected from modification by proteases involved in SFTPB maturation (33). The better utility of SFTPB in serum EVs than in the serum as a predictive biomarker for ILD progression might be related to differences in SFTPB forms between serum EVs and serum.

Persistent inflammation could cause immune and inflammatory cells to secrete pro-fibrotic molecules and might thus be a cause of fibrosis progression in ILDs (34). Since bleomycin treatment causes pulmonary injury, inflammation, and subsequent fibrosis, bleomycin-induced lung fibrosis models would partially recapitulate the pathogenesis of PPF (35). Here, pro-SFTPB protein levels were transiently increased during the development of bleomycin-induced fibrosis, commonly in lung tissue and serum EVs, suggesting that the dynamics of pro-SFTPB in serum EVs reflect those in fibrotic lung tissues. Furthermore, both SFTPB protein and *Sftpb* mRNA were upregulated during a relatively early phase after bleomycin administration, suggesting that SFTPB upregulation might reflect pro-fibrotic changes rather than completed fibrosis formation. In addition, scRNA-Seq suggested that upregulation of *Sftpb* mRNA could be involved in fibrosis-related pathways such as lipid-related pathways and AMPK signaling pathway (20–22). While *Sftpd* showed similar behavior to *Sftpb*, *Sftpa1* and *Sftpc* behaved differently upon bleomycin administration. As surfactant insufficiency or altered composition is associated with lung diseases, such as acute respiratory distress syndromes and IPF (36, 37), functional differences and interactions among individual surfactant proteins in PPF warrant further investigation.

Thus, our study indicated the associations of SFTPB with the pathogenesis of pulmonary fibrosis. Additionally, other identified biomarkers such as BPI fold containing family B member 1 and periostin, the levels of which were increased in serum EVs in PPF, have been reported to be involved in the pathogenesis of pulmonary fibrosis (38, 39). Furthermore, since angiopoietin-1 and ribosomal protein S5, the levels of which were decreased in serum EVs in PPF, have been reported to be involved in the attenuation of fibrosis in the kidney and heart, respectively (40, 41), decreases in these proteins may contribute to the development of pulmonary fibrosis. Thus, proteomics of serum EVs could be applied to not only discover clinical biomarkers but also elucidate mechanisms of pathogenesis, leading to potential therapeutic approaches.

Despite the notable advantages, our study has certain limitations. First, in the discovery cohort, some candidate biomarkers may be missed owing to the low detection sensitivity due to the relatively small sample size. However, our study was able to detect several candidate biomarkers with significant variability despite the small sample size, and among them, SFTPB in serum EVs was verified to be associated with ILD progression using validation cohorts and experiments of human samples and a mouse model. Second, the quantification of biomarkers in serum EVs is currently challenging for clinical applications because of technical problems, such as the complexity of EV isolation, variability in purity and recovery, and difficulty in measuring small protein quantities in EVs. However, advanced technologies, such as digital ELISA with high sensitivity, have emerged (42). Third, the association of SFTPB in serum EVs with life expectancy was limited to cases with an ILD-GAP index of 3 or less. This may be explained by the effects of small sample size and the possibility that in severe cases with a high ILD-GAP index, complications such as infection may have a greater impact on life expectancy than the progression of ILD itself, unlike in mild cases. Meanwhile, unlike SFTPB in serum EVs, serum SFTPB has not been shown to be useful for predicting ILD progression. Nevertheless, serum SFTPB has demonstrated significant disease specificity in ILD. Further validation with awareness of each form of SFTPB in serum is warranted. Finally, we did not account for ILD therapeutics, such as corticosteroids and antifibrotic agents, in this study. Further validation of the impact of such agents on the levels and performance of the candidate biomarkers is needed.

In conclusion, proteomic analyses of serum EVs identified SFTPB as a biomarker for predicting the progression of non-IPF-ILD. Our study suggested that SFTPB in serum EVs consisting mainly of its pro-form could be helpful for predicting non-IPF-ILD progression, unlike SFTPB in serum. As shown in our study, a systems biology platform consisting of clinical data, omics, and bioinformatics can lead to a better understanding of the complexity of PPF and enable the development of personalized medicine.

## Methods

### Sex as a biological variable.

Our study examined male and female mice. The effect of sex differences on the findings is unknown.

### Study population.

Fifty-six PPF cases, 86 non-PPF cases, and 34 HCs in the Osaka University Hospital were included in the discovery cohort. We evaluated the variability of serum EV proteins in these cases to search for PPF biomarker candidates. We then added 78 non-IPF-ILD cases that could not be evaluated for PPF or non-PPF because of missing data of the patients to assess the clinical features of the biomarker candidates.

Next, 2,483 patients whose serum samples were collected from January 1, 2013, to March 31, 2021, and stored at –80°C in the Osaka University Hospital, were reviewed. Fourteen PPF cases, 20 non-PPF cases, and 23 HCs, which were not included in the discovery cohort, were identified and constituted the validation cohort. In the latter, we analyzed the proteomics data of serum EVs from these patients for biomarker evaluation.

Furthermore, in the combined-sample cohort, the serum levels of identified biomarker proteins were measured in 212 available ILD cases, 49 available HCs in the above 2 independent cohorts, and 77 cases with other respiratory diseases (20, BA; 20, COPD; 22, lung cancer; and 15, nontuberculous mycobacterial infection) using a commercial ELISA kit. The correlations between these serum levels and corresponding clinical features were evaluated.

All ILD cases were diagnosed through multidisciplinary discussion based on the American Thoracic Society and European Respiratory Society guidelines (2, 5). ILD cases with acute exacerbations were excluded. The diagnosis of PPF and non-PPF was based on a modified definition from the INBUILD study as shown below.

### Assessment of ILDs.

Based on a modified definition from the INBUILD study (6), PPF was defined as a case with non-IPF-ILD that met 1 of the following criteria within 24 months postdiagnosis despite meeting the standard of care: (i) a relative decline in %FVC ≥ 10%, (ii) a relative decline in %FVC by 5%–10% and worsening of respiratory symptoms or increased extent of fibrosis on CT images, or (iii) worsening of respiratory symptoms and increased extent of fibrosis on CT images. Non-PPF was defined as a case with non-IPF-ILD that did not meet the above criteria, except for cases that were not evaluated owing to missing data. CT patterns of interstitial lung disease were classified into usual interstitial pneumonia (UIP), probable UIP, indeterminate UIP, and alternative diagnosis based on the American Thoracic Society and European Respiratory Society guidelines (5); alternative diagnosis was further classified into subgroups, such as nonspecific interstitial pneumonia (NSIP), organizing pneumonia, hypersensitivity pneumonitis, and pleuroparenchymal fibroelastosis pattern. The diagnosis of idiopathic NSIP in patients without surgical lung biopsy was based on the absence of underlying disease and the NSIP pattern on CT images. The ILD-GAP index was calculated according to the definition by Ryerson et al. (16).

### Isolation of EVs from serum.

EVs were isolated according to a previously described method (43). Briefly, small phosphatidylserine-positive EVs mainly containing exosomes, which are distinct from microvesicles, were purified from 200 μL of serum using the MagCapture isolation kit (FUJIFILM Wako Pure Chemical Corp.). Our EV isolation and characterizations were performed according to the MISEV2018 guidelines (44).

### Nanoparticle tracking analysis.

Analysis of the EV number and size distribution was performed using the LM10HS with a blue laser system (NanoSight), as previously described (45). Briefly, nanoparticle tracking analysis (NTA) was performed on isolated EVs that were diluted 20-fold with phosphate-buffered saline (PBS). All events were recorded in a 60-second video for further analysis using the NTA software. The Brownian motion of particles was tracked between frames to calculate the size using the Stokes-Einstein equation.

### Proteome analysis.

Each eluate of EVs was supplemented with 10× phase-transfer surfactant buffer comprising 500 mM NH_4_HCO_3_, 120 mM sodium deoxycholate, and 120 mM sodium N-lauryl sarcosinate and boiled at 95°C for 5 minutes. The sample was treated with 10 mM tris(2-carboxyethyl)phosphine for 30 minutes at 37°C and then alkylated with 20 mM iodoacetamide for 30 minutes at 37°C in the dark, followed by digestion with 2 mAU LysC (Wako-Chemical) and 1 μg trypsin (Wako-Chemical) at 37°C overnight. The digested solutions were acidified with 1% trifluoroacetic acid (TFA) and centrifuged at 20,000*g* for 10 minutes at room temperature to precipitate the detergents. Supernatants containing digested peptides were desalted using a C18-SCX StageTip and dried using a centrifugal evaporator. The dried peptides were dissolved in 2% acetonitrile (ACN) and 1% TFA.

LC-MS/MS was performed by coupling an UltiMate 3000 Nano LC system (Thermo Fisher Scientific) and an HTC-PAL autosampler (CTC Analytics) to an Orbitrap Fusion Lumos mass spectrometer (Thermo Fisher Scientific). The peptides were delivered to an analytical column (75 μm × 20 cm, packed in-house with ReproSil-Pur C18-AQ, 1.9 μm resin; Dr. Maisch, Germany) and separated at a flow rate of 280 nL/min using a 45-minute gradient from 5% to 30% of solvent B (solvent A, 0.1% formic acid [FA]; solvent B, 0.1% FA and 99.9% ACN). The Orbitrap Fusion Lumos mass spectrometer was operated in the 5 gas-phase fractionation–DIA (GPF-DIA) mode (120,000 precursor resolution, 50,000 fragment resolution, automatic gain control [AGC] target of 1,000,000 and 200,000 for MS1 and MS2, maximum ion inject time [max IIT] of 250 ms and 86 ms for MS1 and MS2, normalized collision energy [NCE] of 30, and *m/z* 2 precursor isolation window) covering *m/z* 418–494, 490–566, 562–638, 634–710, and 706–782 (5× GPF). Individual samples were analyzed by the DIA mode (120,000 precursor resolution, 30,000 fragment resolution, AGC target of 400,000 and 200,000 for MS1 and MS2, max IIT of 100 ms and 54 ms for MS1 and MS2, NCE of 30, and *m/z* 8 precursor isolation window). The DIA data were analyzed using Spectronaut 15, which was configured to use the default settings of library-based DIA analysis. The search results were quantified and filtered to a 1% precursor level. The MS files were searched against a UniProt human database. Run-wise imputation was performed for missing values. One commercial serum sample was added to every 15 samples as a quality control to ensure that the quality was retained from sample preparation to data analysis. DIA analysis of digested HeLa cells (Thermo Fisher Scientific) was also performed as a quality control for MS analysis. The proteomics abundance data were normalized using the variance stabilization normalization method (46), implemented using the limma package in R studio (version 4.0.3).

### ELISA using commercially available kits.

The serum levels of SFTPB were measured using the SEB622Hu ELISA kits (Cloud-Clone Corp.). The immunogen for the ELISA kit SEB622Hu was Phe201~Leu381, and the antibodies in this kit were polyclonal antibodies. Therefore, the antibodies can react with several forms of SFTPB, such as pro-SFTPB, Cpro-SFTPB, and mature SFTPB.

### Immunohistochemistry analysis.

Lung tissue samples were obtained from patients with or without PPF who underwent surgery for suspected lung cancer. Specimens were prepared from areas without tumor lesions. Paraffin-fixed tissues were deparaffinized using xylene and alcohol, incubated with 10 mmol/L citrate buffer (pH 6.0) for antigen retrieval, oxidized using 3% hydrogen peroxide at room temperature for 10 minutes, and then blocked with 3% bovine serum albumin in PBS at room temperature for 1 hour. The slides were incubated with anti-SFTPB antibodies (1:20,000; sc-133143; Santa Cruz Biotechnology), followed by incubation with horseradish peroxidase–conjugated anti-mouse (414132F; Nichirei Biosciences) secondary antibody at room temperature for 30 minutes. Image acquisition was performed using an Olympus BX51 microscope.

### Western blotting analysis.

To examine the levels of SFTPB in lung tissues, serum, serum EVs, and BALF in patients with PPF and bleomycin-induced pulmonary fibrosis model mice compared with those in controls, we performed Western blotting. Protein samples were loaded onto NuPAGE 4%–12% or 12% Bis-Tris gels (Thermo Fisher Scientific). For immunoblot analysis, the gels were electroblotted onto polyvinylidene difluoride membranes (Bio-Rad). Membranes were blocked with Blocking One (Nacalai Tesque) at room temperature for 30 minutes, incubated with the specific primary antibody (Supplemental Table 11), and then incubated with the appropriate secondary antibody. The immunoreactive signals were visualized using SuperSignal West Atto Ultimate Sensitivity Chemiluminescent Substrate (Thermo Fisher Scientific) or Chemi-Lumi One Super (Nacalai Tesque) and detected with an ImageQuant LAS500 system (GE Healthcare, now Cytiva). Thereafter, for lung tissue specimens, the blots were stripped using Western blot Stripping Solution (Nacalai Tesque) under gentle shaking at room temperature for 15 minutes, followed by probing of the blots using an antibody against the loading control protein β-actin. Band intensities were quantified using the ImageJ software (NIH, version 1.53k). In lung tissue specimens, SFTPB levels were normalized to the level of β-actin. Serum and serum EV samples per lane were standardized based on the amount of source serum, while BALF samples per lane were standardized based on the amount of source BALF.

### Bleomycin-induced pulmonary fibrosis mouse model.

For immunochemistry and Western blotting analysis, we used 7-week-old mice (C57BL/6J strain, CLEA Japan) that were bred at specific pathogen–free facilities at Osaka University. These mice were anesthetized with isoflurane and administered a single endotracheal dose of bleomycin (5 mg/kg body weight) on day 0. Bleomycin-naive mice (control) were euthanized on day 0, whereas bleomycin-treated mice were euthanized on days 3, 10, and 21 for collecting lung tissues, BALF, serum, and serum EVs (*n* = 3–5 mice per group). For scRNA-Seq analysis of the lungs, we used 8-week-old C57BL/6J mice (Sankyo Labo Service Corporation) that were bred at specific pathogen–free facilities at Tokyo University of Science. These mice were anesthetized with isoflurane and administered a single endotracheal dose of bleomycin (1.25 mg/kg body weight) on day 0, whereas bleomycin-treated mice were euthanized on days 3, 7, 14, and 28 to collect lung tissues (*n* = 5 mice per group).

### Single-cell analysis using bleomycin-induced pulmonary fibrosis model mice.

Single-cell suspensions of the lungs of bleomycin-treated and control mice were prepared as described previously (47). The resultant single-cell suspensions were stained by SampleTag antibodies (BD Biosciences) and pooled; scRNA-Seq libraries were prepared using a Rhapsody system (BD Biosciences) and terminator-assisted solid-phase cDNA amplification and sequencing (TAS-Seq) protocol (47). Sequencing was performed using the Illumina NovaSeq 6000 sequencer (Illumina) and NovaSeq 6000 S4 Reagent Kit v1.5 (200 cycles) (Illumina) following the manufacturer’s instructions (read1 67 base pairs [bp], read2 151 bp, index1 8 bp, index2 8 bp). The TAS-Seq data processing and demultiplexing by SampleTag expression were performed as described previously (47). Cell clustering of the data was performed using Seurat v4 as described previously (47); each cell cluster was annotated by its expression pattern of marker genes (Supplemental Table 12). The data set comprised 5 untreated control mice and 5 mice each on days 3, 7, 14, and 28 after bleomycin treatment. Pseudo-bulk data were constructed using a method described by Piper et al. (48). Sftpb coexpression analysis was performed using hdWGCNA, which is a WGCNA algorithm optimized for single-cell sparse data (19). GO (biological process) and KEGG pathway enrichment analyses of a group of genes in the same module as Sftpb were performed using Metascape (14).

### Case-control association test for individual protein abundance.

We performed association tests between individual normalized protein abundance and the disease state using the limma method. Age and sex were also included as covariates. Statistical significance was determined using an FDR *q* < 0.3 and a log_2_FC > 1.0 or < –1.0. Analysis was performed using the limma package in R studio (version 4.0.3).

### Tissue expression profiles of serum EV proteins.

We referred to consensus mRNA expression data from the Human Protein Atlas to investigate the tissue expression profiles of serum EV proteins. To compare the tissue expression profiles among proteins, a heatmap was created using the *Z* scores calculated from the expression levels. The proportions of lung-specific proteins were compared by χ^2^ analysis. Lung-specific proteins were defined as having a *Z* score higher than 2.

### Statistics.

Biomarker protein abundances were compared based on conditions, imaging findings, and respiratory function using 1-way ANOVA, and Bonferroni’s or Holm’s method was applied to adjust the obtained ANOVA *P* values. Differences in means between 2 groups were compared using 2-tailed Student’s *t* test. The relationship between the levels of biomarkers and respiratory function was analyzed using Pearson’s correlation analysis.

In patients with INSIP, CVD-ILD, FHP, and unclassifiable ILD, which represent PPF, ROC analysis was performed to evaluate the utility for predicting ILD progression. ILD progression was defined as death within 1 year, acute exacerbation within 1 year, or at least 10% decrease in %FVC within 1 year. Composite biomarkers in ROC analysis were analyzed using logistic regression models.

Similarly, in patients with INSIP, CVD-ILD, FHP, and unclassifiable ILD, the OS was estimated using the Kaplan-Meier method, and the log-rank test was used to assess differences between 2 comparison groups. Univariate and multivariate Cox proportional hazard regression models were adopted to determine HRs. Given that the ILD-GAP index did not satisfy the proportional hazard assumption, stratified proportional hazards model by ILD-GAP index was used to test whether SFTPB level in serum EVs was an independent prognostic factor from ILD-GAP index. OS was defined as the period from the date of blood collection to the date of death from any cause. Data for patients not reported as deceased at the time of analysis were censored on the date that they were last known to be alive.

The final analysis was conducted on April 27, 2022, for the discovery cohort and on August 31, 2022, for the validation cohort using the patients’ medical records.

The above statistical analyses were performed using EZR software, version 1.38. Statistical significance was set at *P* < 0.05.

### Study approval.

This study was conducted according to the Declaration of Helsinki for medical research involving human participants and was approved by the Osaka University Hospital Ethics Committee (approval number: 17148). All animal experiments were reviewed and approved by the Animal Experiment Committee of Osaka University (approval number:01-021-016) and Tokyo University of Science (approval numbers: S17034, S18029, S19024, and S20019). Written informed consent was obtained from all patients before enrollment in this study.

### Data availability.

scRNA-Seq data obtained using bleomycin-induced pulmonary fibrosis model mice in our study are available via the National Center for Biotechnology Gene Expression Omnibus with the accession code GSE264278.

## Author contributions

TE, Y Shirai, YT, and AK designed the study, analyzed the data, and wrote the manuscript. RE, SS, M Nakayama, MTI, Y Noda, YA, T Kawasaki, T Koba, YF, M Yaga, YH, HY, SA, RH, M Yamamoto, DN, Y Suga, M Naito, K Masuhiro, HH, KI, IN, K Miyake, SK, KF, TS, Y Naito, SF, YNK, SN, M Yanagawa, Y Shintani, MNI, KM, JA, TT, and YI contributed to data acquisition and interpretation and participated in revising the manuscript critically. All authors approved the final version of the manuscript for submission. The authorship order among the 2 co–first authors was determined by discussion among all authors.

## Supplementary Material

Supplemental data

Unedited blot and gel images

Supporting data values

## Figures and Tables

**Table 2:**
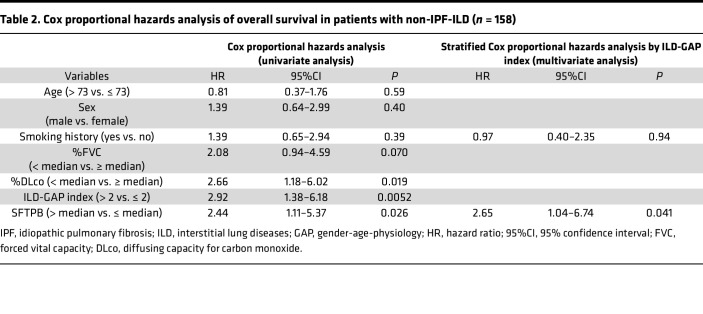
Cox proportional hazards analysis of overall survival in patients with non-IPF-ILD (*n* = 158)

**Table 1:**
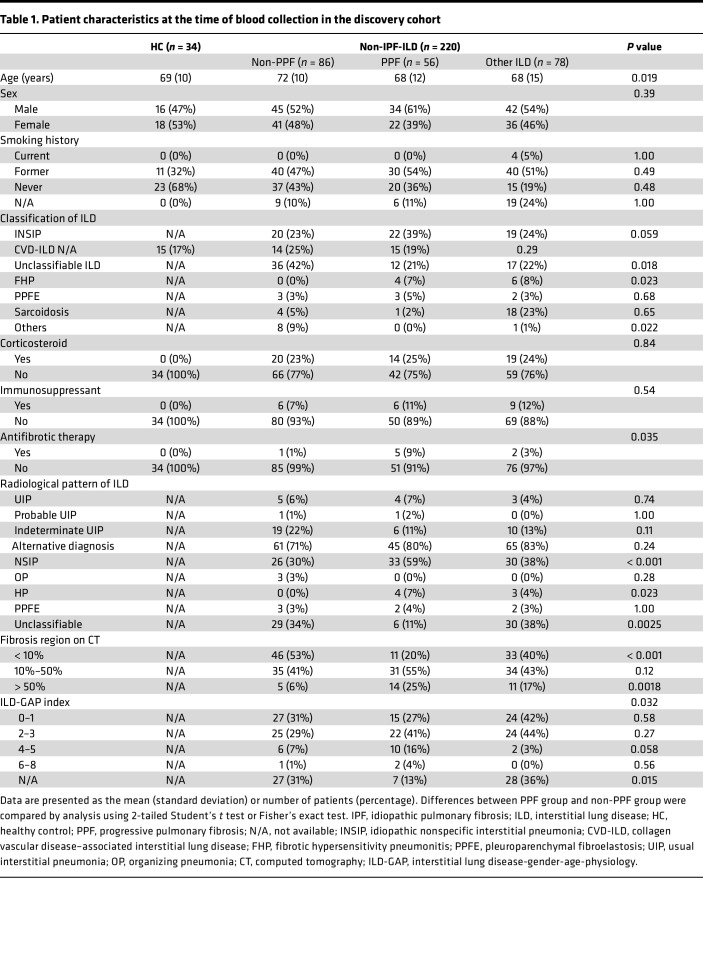
Patient characteristics at the time of blood collection in the discovery cohort

## Version 1 06/10/2024

Electronic publication
