# Supplementary material for: SFTPB in serum extracellular vesicles as a biomarker of progressive pulmonary fibrosis
Source: JCI Insight. 2024 Jun 10;9(11):e177937. doi: 10.1172/jci.insight.177937 (PMC11382876; doi:10.1172/jci.insight.177937)
Supplement: Supplemental data [file jciinsight-9-177937-s165.pdf]

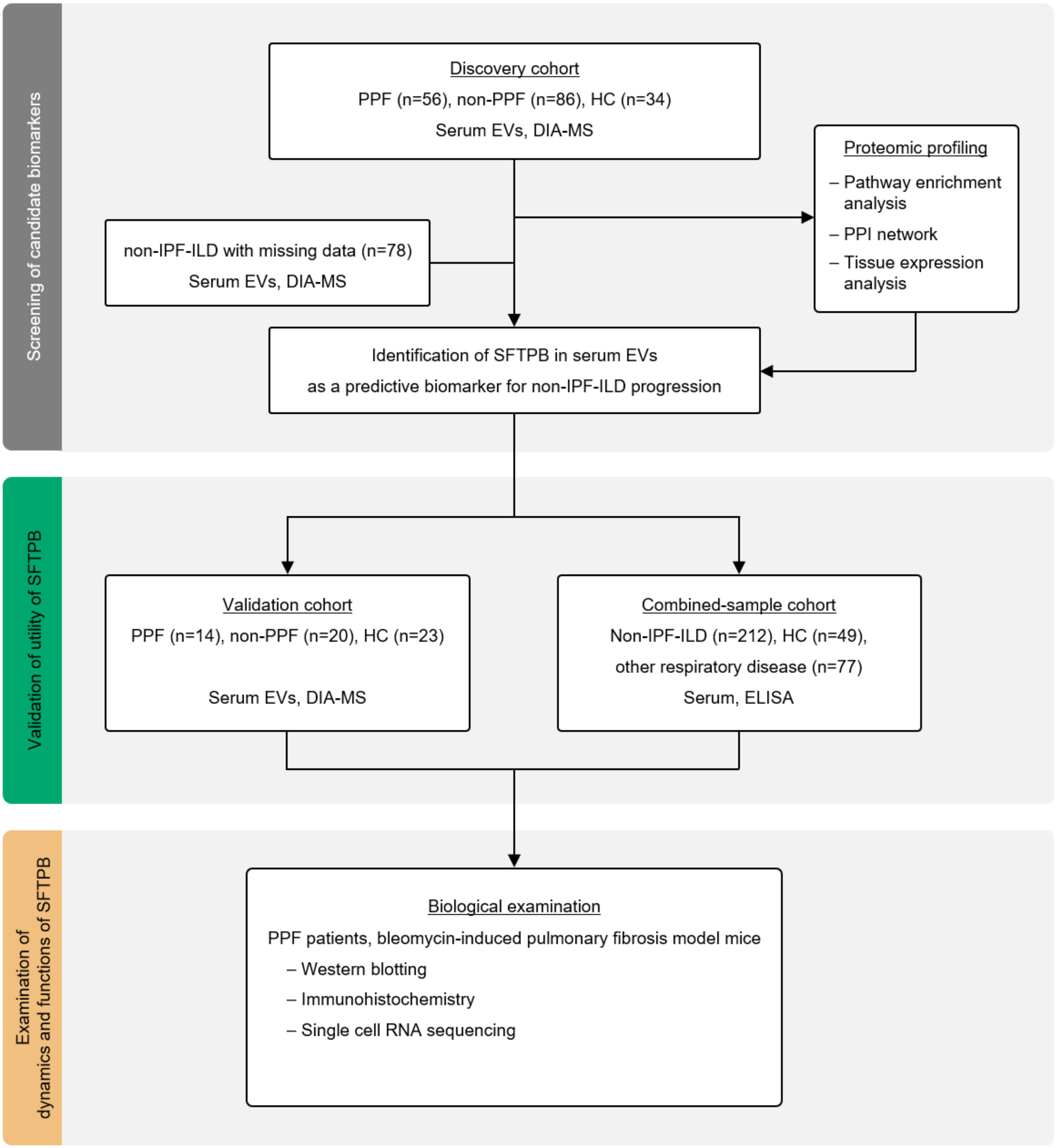

Supplemental Figure 1. A flowchart of this study

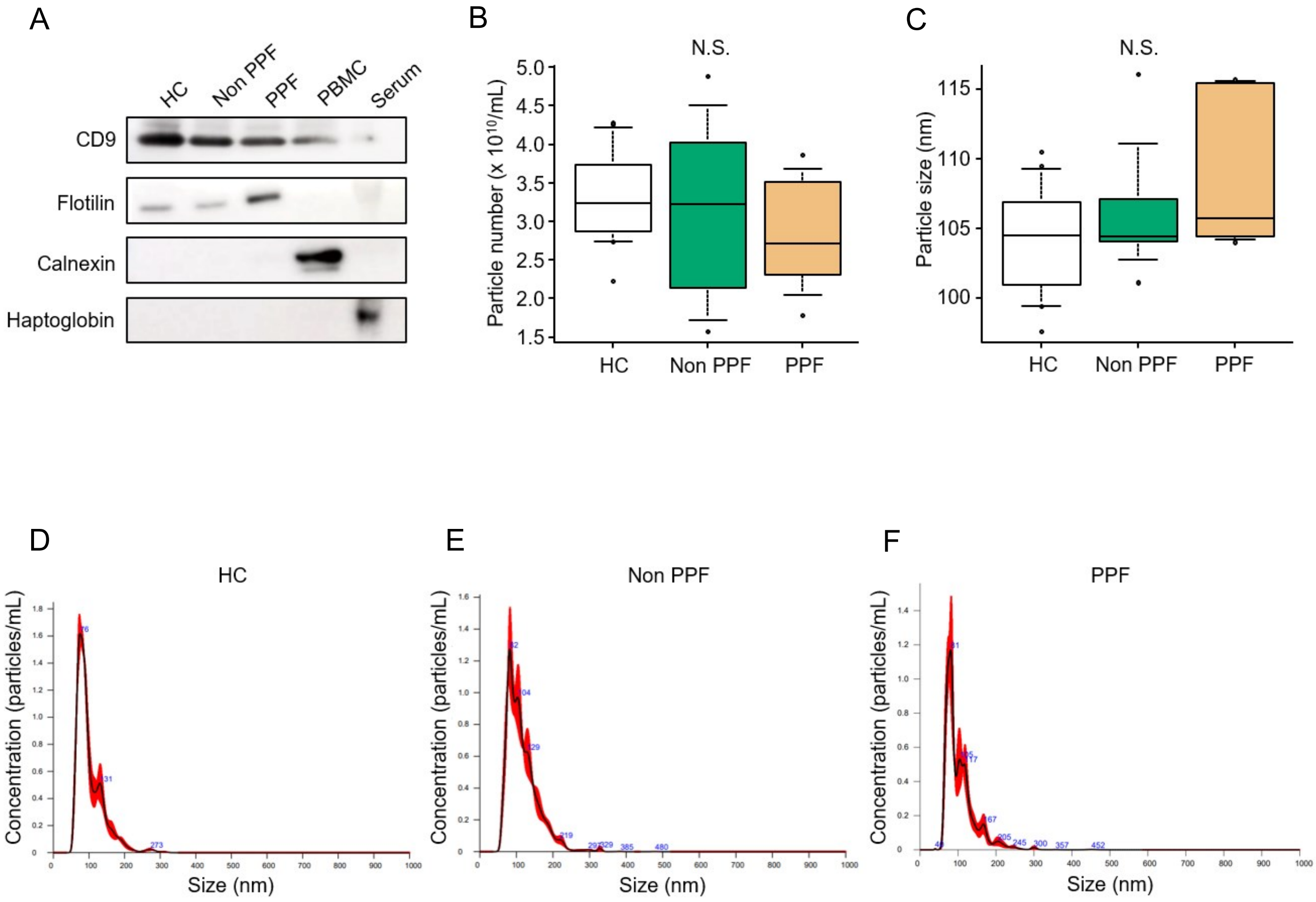

Supplemental Figure 2. Characterization and quantification of serum extracellular vesicles

(A) Immunoblots comparing CD9, flotillin, calnexin, and haptoglobin levels in serum EVs vs. peripheral blood mononuclear cells (PBMCs) and serum. (B, C) Boxplots showing the mean diameters and numbers of serum EVs analyzed using NanoSight from healthy controls (HCs) (n = 12), non-PPF (n = 7), and PPF cases (n = 6). The data were subjected to analysis of variance, and the Holm’s method was applied to adjust for *P* values. The boxes indicate interquartile ranges (75% and 25%) and medians; the upper and lower whiskers represent the 10% and 90% points, respectively. (D–F) Representative figures of the particle size distribution curve of serum EVs from a healthy control (D), a non-progressive pulmonary fibrosis (non-PPF) case (E), and a PPF case (F) using NanoSight.

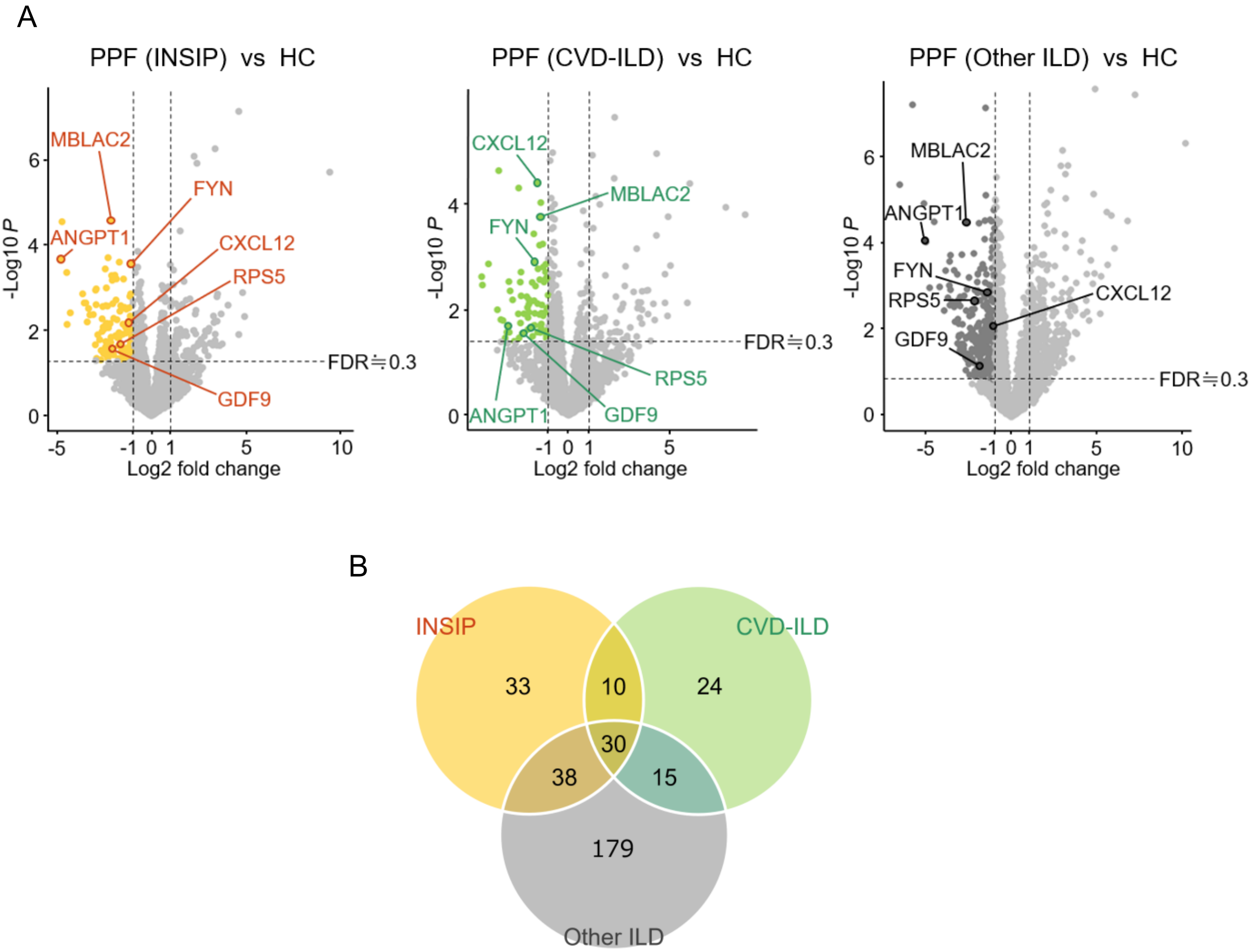

Supplemental Figure 3. Serum EV proteins, the levels of which were decreased in the progressive pulmonary fibrosis group compared with those in healthy controls

(A, B) Identification of proteins in serum EVs, the levels of which were decreased in the three progressive pulmonary fibrosis groups—idiopathic nonspecific interstitial pneumonia (n = 22), collagen vascular disease-associated interstitial lung disease (n = 14), and other interstitial lung disease (n = 20)—compared with those in healthy controls (n = 34).

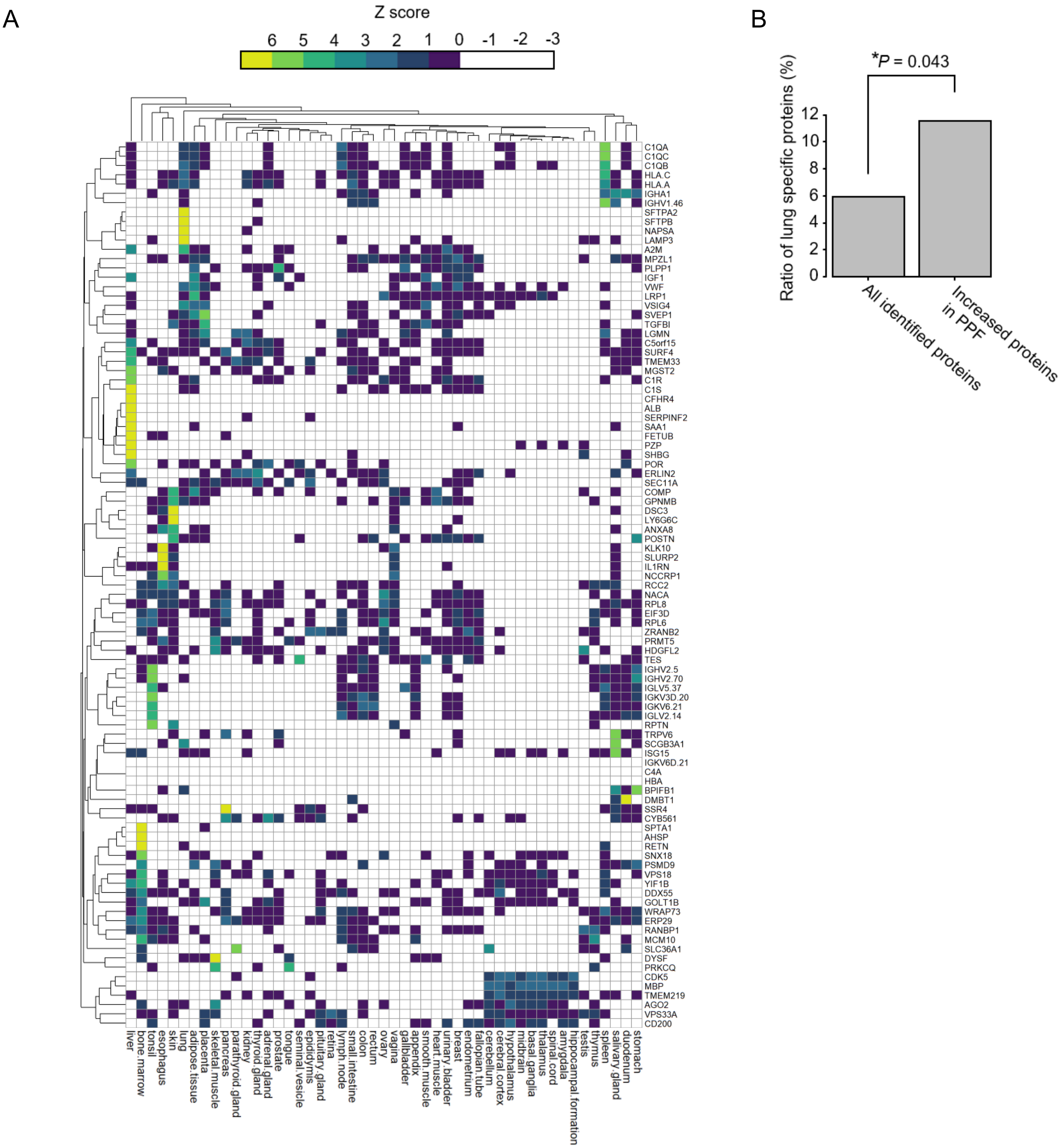
A
Z score
6 5 4 3 2 1 0 -1 -2 -3
C1QA
C1QC
C1QB
HLA.C
HLA.A
IGHA1
IGHV1.46
SFTPA2
SFTPB
NAPSA
LAMP3
A2M
MPZL1
PLPP1
IGF1
VWF
LRP1
VSIG4
SVEP1
TGFBI
LGMN
C5orf15
SURF4
TMEM33
MGST2
C1R
C1S
CFHR4
ALB
SERPINF2
SAA1
FETUB
PZP
SHBG
POR
ERLIN2
SEC11A
COMP
GPNMB
DSC3
LY6G6C
ANXA8
POSTN
KLK10
SLURP2
IL1RN
NCCRP1
RCC2
NACA
RPL8
EIF3D
RPL6
ZRANB2
PRMT5
HDGFL2
TES
IGHV2.5
IGHV2.70
IGLV5.37
IGKV3D.20
IGKV6.21
IGLV2.14
RPTN
TRPV6
SCGB3A1
ISG15
IGKV6D.21
C4A
HBA
BPIFB1
DMBT1
SSR4
CYB561
SPTA1
AHSP
RETN
SNX18
PSMD9
VPS18
YIF1B
DDX55
GOLT1B
WRAP73
ERP29
RANBP1
MCM10
SLC36A1
DYSF
PRKCQ
CDK5
MBP
TMEM219
AGO2
VPS33A
CD200
liver
bone.marrow
tonsil
esophagus
skin
lung
adipose.tissue
placenta
skeletal.muscle
pancreas
parathyroid.gland
kidney
thyroid.gland
adrenal.gland
prostate
tongue
seminal.vesicle
epididymis
pituitary.gland
retina
lymph.node
small.intestine
colon
rectum
ovary
vagina
gallbladder
appendix
smooth.muscle
heart.muscle
urinary.bladder
breast
endometrium
fallopian.tube
cerebellum
cerebral.cortex
hypothalamus
midbrain
basal.ganglia
thalamus
spinal.cord
amygdala
hippocampal.formation
testis
thymus
spleen
salivary.gland
duodenum
stomach
B
*P = 0.043
Ratio of lung specific proteins (%)
0 2 4 6 8 10 12
All identified proteins
Increased proteins in PPF

Supplemental Figure 4. Tissue expression profiles of progressive pulmonary fibrosis biomarker candidates, the levels of which were increased in serum EVs

(A) Heatmap representing the levels of tissue-specific mRNA expression of 95 biomarker candidates commonly increased in at least two progressive pulmonary fibrosis subgroups compared with those in healthy controls, based on tissue mRNA consensus gene data of the Human Protein Atlas. The expression levels are indicated by a Z score. Expression levels of IGKV6D-2, C4A, and HBA were unavailable (Z score = 0). (B) Rate of lung-specific proteins among all identified proteins as well as among biomarker candidate proteins in (A). These biomarker candidates were enriched for lung-specific proteins (percentage of lung-specific proteins: 5.95% vs. 11.58%, P = 0.043 by chi-squared test).

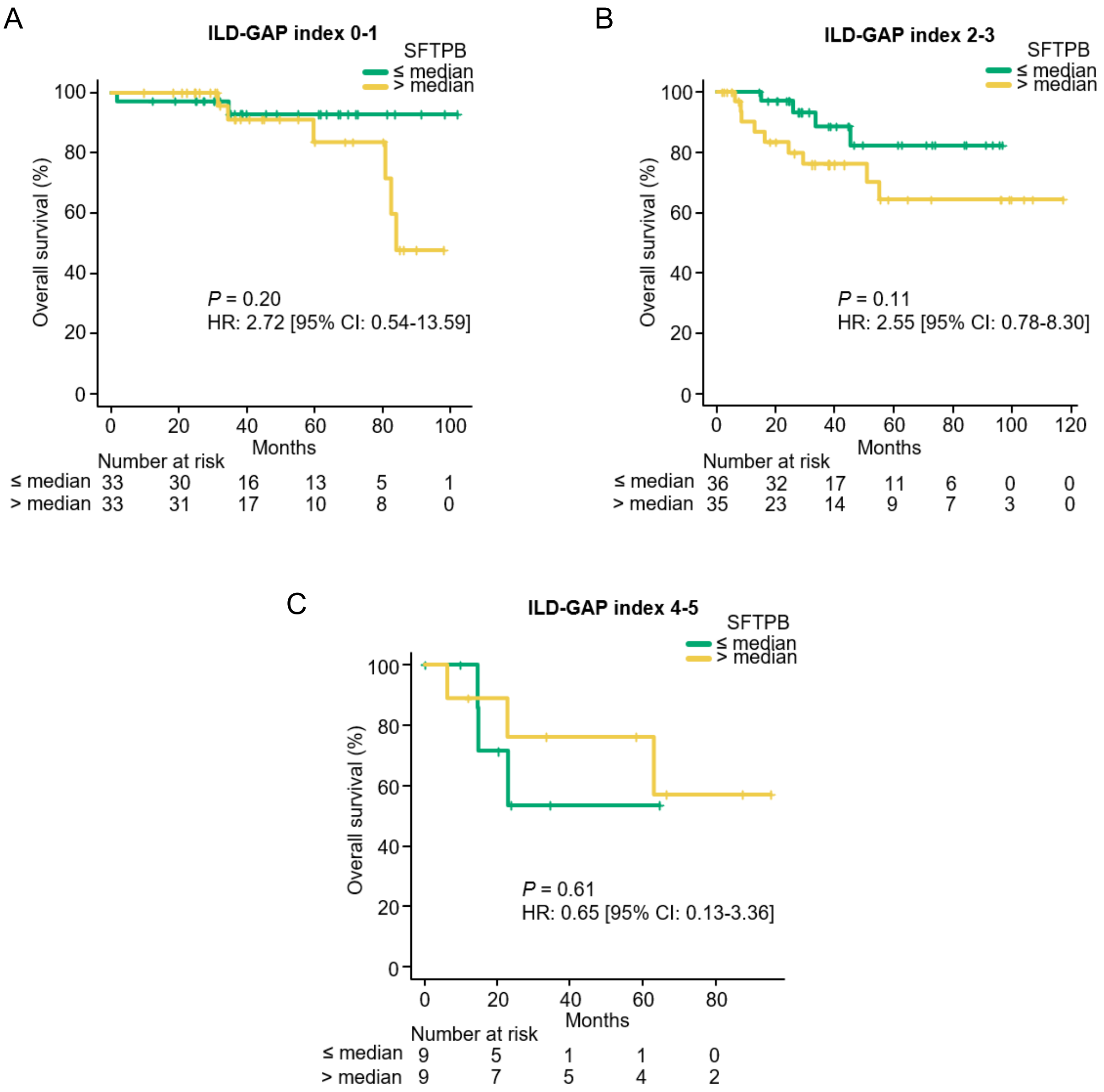

Supplemental Figure 5. Subgroup analysis of the ILD-GAP index using the Kaplan–Meier curve of SFTPB levels in serum EVs in the discovery cohort
(A) Subgroup analysis of the ILD-gender age physiology (ILD-GAP) index 0–1 using the Kaplan–Meier curve of SFTPB levels in serum EVs (hazard ratio [HR] 2.72; 95% confidence interval [CI] 0.54–13.59); P = 0.20 by log-rank test). (B) Subgroup analysis of the ILD-GAP index 2–3 (HR 2.55; 95% CI 0.78–8.30); P = 0.11 by log-rank test). (C) Subgroup analysis of the ILD-GAP index 4–5 (HR 0.65; 95% CI 0.13–3.36); P = 0.61 by log-rank test).

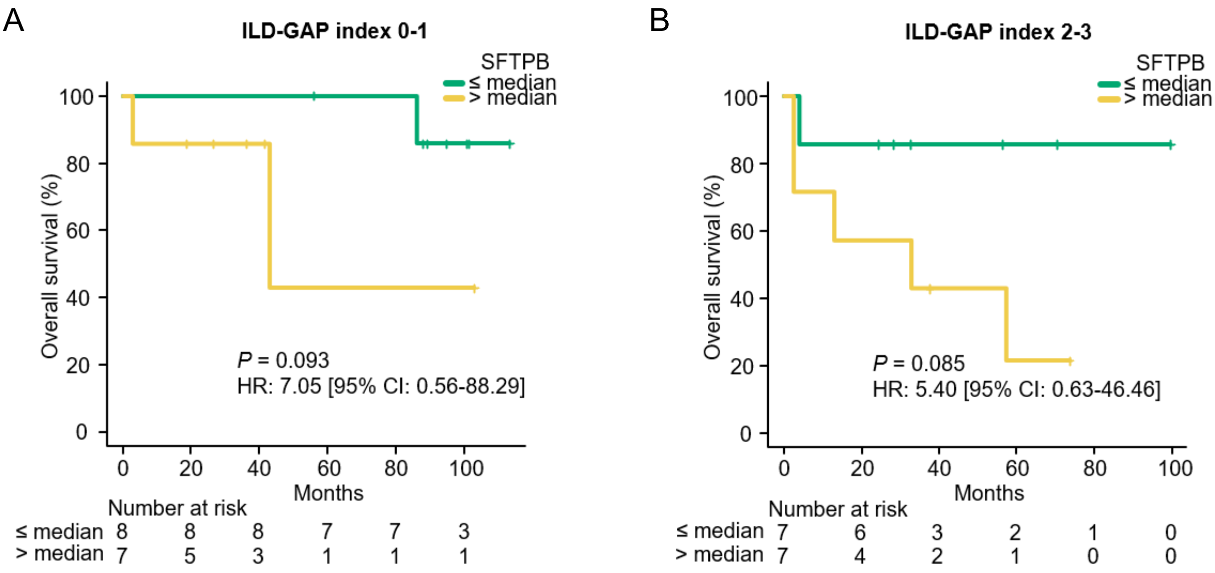

Supplemental Figure 6. Subgroup analysis of the ILD-GAP index using the Kaplan–Meier curve of SFTPB levels in serum EVs in the validation cohort
(A) Subgroup analysis of the ILD-GAP index 0-1 (HR: 7.05 (95% CI: 0.56-88.29), P = 0.093 by log-rank test). (B) Subgroup analysis of the ILD-GAP index 2-3 (HR: 5.40 (95% CI: 0.63-46.46), P = 0.085 by log-rank test).

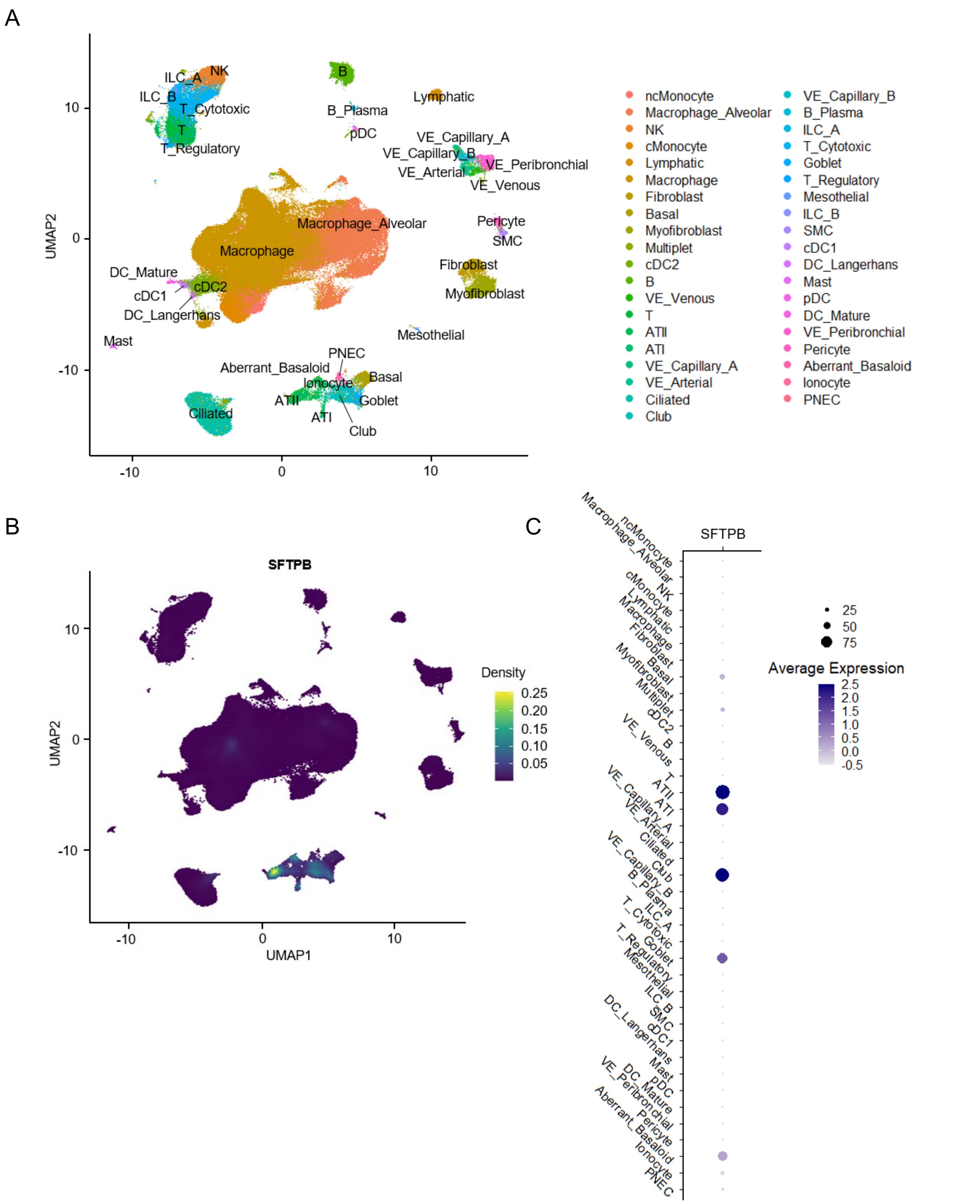
A
B
C
SFTPB
UMAP1
UMAP2
Density
Average Expression
Macrophage_Alveolar
Macrophage
Ciliated
Fibroblast
Myofibroblast
Mesothelial
Lymphatic
Pericyte
SMC
Mast
PNEC
Aberrant_Basaloid
Ionocyte
Basal
ATII
ATI
Goblet
Club
NK
ILC_A
ILC_B
T_Cytotoxic
T
T_Regulatory
B
B_Plasma
pDC
VE_Capillary_A
VE_Capillary_B
VE_Peribronchial
VE_Arterial
VE_Venous
DC_Mature
cDC1
cDC2
DC_Langerhans
ncMonocyte
cMonocyte
Multiplet

Supplemental Figure 7. Analysis of SFTPB mRNA expression of previously published singe-cell transcriptomes from control and idiopathic pulmonary fibrosis lungs

(A) UMAP embedding of single-cell RNA sequencing data from control and idiopathic pulmonary fibrosis lungs. (B) Density plots of the mRNA expression levels of SFTPB. (C) Dot plots of the mRNA expression levels of SFTPB.

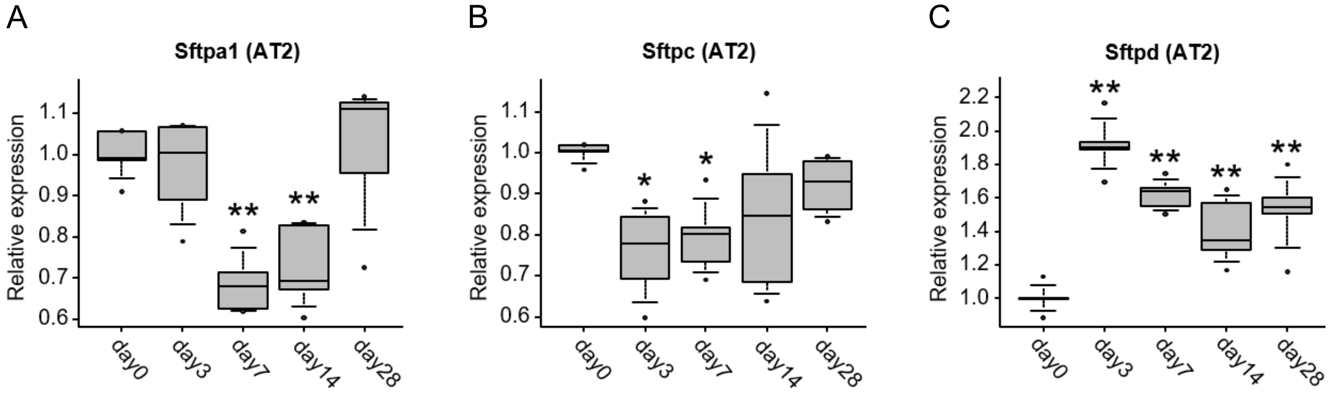

Supplemental Figure 8. Single-cell RNA sequencing in a bleomycin-induced pulmonary fibrosis mouse model
(A, B, C) Pseudo-bulk analysis of the expression of Sftpa1, Sftpc, and Sftpd in alveolar epithelial type 2 cells (ATII). The boxes indicate interquartile ranges (75% and 25%) and medians; the upper and lower whiskers represent the 10% and 90% points, respectively. The expression levels were compared by analysis of variance (ANOVA), and the Dunnett's method was applied to adjust for the ANOVA P values. *, $P < 0.05$; **, $P < 0.01$. n = 5 mice per group.

Supplemental Table 1. Patient characteristics at the time of blood collection in the validation cohort

	HC‡ (n = 23)	Non-IPF*-ILD† (n = 34)		P-value
		Non-PPF§ (n = 20)	PPF (n = 14)	
Age (years)	70 (12)	67 (12)	58 (12)	0.040
Sex				1.00
Male	12 (52%)	8 (40%)	6 (43%)	
Female	11 (48%)	12 (60%)	8 (57%)	
Smoking history				
Current	3 (13%)	0 (0%)	1 (7%)	0.41
Former	7 (30%)	9 (45%)	7 (79%)	1.00
Never	12 (52%)	7 (35%)	5 (36%)	1.00
N/A‖	1 (4%)	4 (20%)	1 (7%)	0.38
Classification of ILD				
INSIP**	N/A	0 (0%)	1 (7%)	0.41
CVD-ILD††	N/A	16 (80%)	6 (43%)	0.036
Unclassifiable ILD	N/A	3 (15%)	5 (36%)	0.23
FHP‡‡	N/A	0 (0%)	0 (0%)	1.00
PPFE§§	N/A	0 (0%)	2 (14%)	0.16
Sarcoidosis	N/A	0 (0%)	0 (0%)	1.00
Others	N/A	1 (5%)	0 (0%)	1.00
Corticosteroid				1.00
Yes	0 (0%)	10 (50%)	7 (50%)	
No	23 (100%)	10 (50%)	7 (50%)	
Immunosuppressant				0.74
Yes	0 (0%)	10 (50%)	6 (43%)	
No	23 (100%)	10 (50%)	8 (57%)	
Antifibrotic therapy				0.41
Yes	0 (0%)	0 (0%)	1 (7%)	
No	23 (100%)	20 (100%)	13 (93%)	
Radiological pattern of ILD				
UIP‖‖	N/A	2 (10%)	2 (14%)	1.00
Probable UIP	N/A	2 (10%)	0 (0%)	0.50
Indeterminate UIP	N/A	10 (50%)	1 (7%)	0.011
Alternative diagnosis	N/A	6 (30%)	11 (79%)	0.013
NSIP	N/A	4 (13.3%)	1 (7%)	0.38
OP***	N/A	0 (0%)	0 (0%)	1.00
HP	N/A	0 (0%)	0 (0%)	1.00
PPFE	N/A	0 (0%)	2 (14%)	0.16
Unclassifiable	N/A	2 (0%)	8 (57%)	0.0061
Fibrosis region on CT†††				
< 10%	N/A	10 (50%)	1 (7%)	0.011
10–50%	N/A	7 (35%)	7 (50%)	0.49
> 50%	N/A	3 (15%)	6 (43%)	0.12
ILD-GAP‡‡‡ index				
0–1	N/A	11 (55%)	4 (29%)	0.17
2–3	N/A	7 (35%)	7 (50%)	0.49
4–5	N/A	1 (5%)	0 (0%)	1.00
> 5	N/A	0 (0%)	1 (7%)	0.41
N/A	N/A	1 (5%)	2 (14%)	0.56

Data are presented as the mean (±standard deviation) or number of patients (percentage). *P*-values were calculated between PPF group and non-PPF group using Student's t-test or Fisher's exact test.

*idiopathic pulmonary fibrosis; †interstitial lung disease; ‡healthy control; §progressive pulmonary fibrosis; ‖not available; **idiopathic nonspecific interstitial pneumonia; ††collagen vascular disease-interstitial lung disease; ‡‡fibrotic hypersensitivity pneumonitis; §§pleuroparenchymal fibroelastosis; ‖‖usual interstitial pneumonia; ***organizing pneumonia; †††computed tomography; ‡‡‡interstitial lung disease-gender age physiology

Supplemental Table 2. List of differentially expressed proteins between progressive pulmonary fibrosis (PPF) and non-PPF in serum extracellular vesicles

UniProtKB/ Swiss-Prot	Symbol	Description	$\log_2$ fold change	$-\log_{10}P$-value
P01764	IGHV3-23	Immunoglobulin heavy variable 3-23	2.89	3.67
*Q07954	*LRP1	*Pro low-density lipoprotein receptor-related protein 1	1.06	3.62
*Q8TDL5	*BPIFB1	*BPI fold containing family B member 1	1.07	3.34
*P07988	*SFTPB	*Surfactant protein B	1.08	3.30
P23246; Q15233	SFPQ; NONO	Splicing factor, proline- and glutamine-rich; non-POU domain-containing octamer-binding protein	3.48	3.23

*included in the 29 proteins that were increased in all three PPF groups (idiopathic nonspecific interstitial pneumonia, collagen vascular disease-interstitial lung disease, and other interstitial lung disease) compared with the healthy control group

Supplemental Table 3. List of the 29 proteins with levels that increased in common between all three progressive pulmonary fibrosis groups (idiopathic nonspecific interstitial pneumonia, collagen vascular disease-interstitial lung disease, and other interstitial lung disease) compared with those in the healthy control group

UniProtKB/ Swiss-Prot	Symbol	Description	$\log_2$ fold change			$-\log_{10}P$-value		
			INSIP†	CVD-ILD‡	Other ILD	INSIP	CVD-ILD	Other ILD
*P07988	*SFTPB	*Surfactant protein B	4.63	4.37	4.94	7.12	4.98	7.60
Q96RF0	SNX18	Sorting nexin-18	3.38	2.05	3.17	6.25	1.57	4.23
Q96QR1	SCGB3A1	Secretoglobin family 3A member 1	2.27	2.29	1.99	6.07	5.67	3.08
*Q8TDL5	*BPIFB1	*BPI fold containing family B member 1	2.42	2.26	1.90	5.91	4.51	3.54
Q8NHQ9	DDX55	ATP-dependent RNA helicase DDX55	9.46	8.76	10.27	5.70	3.82	6.35
*Q07954	*LRP1	*Prolow-density lipoprotein receptor-related protein 1	1.73	1.83	1.24	3.15	2.66	1.86
P62917	RPL8	60S ribosomal protein L8	3.51	3.06	2.34	2.85	1.67	1.22
Q14956	GPNMB	Transmembrane glycoprotein NMB	3.48	3.48	3.83	2.79	2.10	3.11
Q99735	MGST2	Microsomal glutathione S-transferase 2	4.07	4.78	3.35	2.59	2.69	1.92
Q9UGM3	DMBT1	Deleted in malignant brain tumors 1 protein	2.36	3.38	2.03	2.47	3.17	1.61
Q9Y3E0	GOLT1B	Vesicle transport protein GOT1B	3.70	6.01	3.76	2.38	4.41	2.46
P01814	IGHV2-70	Immunoglobulin heavy variable 2-70	2.33	2.65	2.43	2.38	2.05	2.49
Q9HD89	RETN	Resistin	4.94	7.80	5.24	2.32	3.96	2.39
Q6ZVX7	NCCRP1	F-box only protein 50	1.32	1.35	2.13	2.23	1.83	4.25
P02686	MBP	Myelin basic protein	3.05	3.94	2.67	2.02	2.38	1.62
Q5BJH7	YIF1B	Protein YIF1B	3.02	3.65	3.36	1.97	2.36	2.44
Q04759	PRKCQ	Protein kinase C theta type	2.26	2.37	2.94	2.00	1.76	2.73
P02747	C1QC	Complement C1q subcomponent subunit C	1.52	1.74	3.34	2.03	1.80	5.83
P09871	C1S	Complement C1s subcomponent	1.35	1.56	2.91	2.01	1.77	5.82
P0DP57	SLURP2	Secreted Ly-6/uPAR domain-containing protein 2	3.39	3.38	7.30	1.91	1.50	7.47
A0A075B6J1	IGLV5-37	Immunoglobulin lambda variable 5-37	2.81	3.58	3.10	1.86	2.14	2.00
P02746	C1QB	Complement C1q subcomponent subunit B	1.45	1.58	3.19	1.86	1.57	5.63
P00736	C1R	Complement C1r subcomponent	1.31	1.63	3.02	1.83	1.82	6.18
P67812	SEC11A	Signal peptidase complex catalytic subunit SEC11A	2.63	4.94	2.95	1.79	3.78	1.96
P02768	ALB	Albumin	3.11	4.20	4.41	1.70	2.12	2.70
Q9H1D0	TRPV6	Transient receptor potential cation channel subfamily V member 6	3.47	4.27	6.86	1.65	1.81	4.54
P01704	IGLV2-14	Immunoglobulin lambda variable 2-14	1.07	1.79	1.03	1.66	1.86	1.29
Q15063	POSTN	Periostin	3.63	4.07	3.58	1.64	1.42	1.35
P49747	COMP	Cartilage oligomeric matrix protein	2.84	3.54	5.63	1.54	1.85	4.75

*included in the 5 proteins that were increased in the progressive pulmonary fibrosis group compared with the non- progressive pulmonary fibrosis group; †idiopathic nonspecific interstitial pneumonia; ‡collagen vascular disease-interstitial lung disease

Supplemental Table 4. List of the 30 proteins with levels that decreased in all three progressive pulmonary fibrosis groups (idiopathic nonspecific interstitial pneumonia, collagen vascular disease-interstitial lung disease, and other interstitial lung disease), compared with those in the healthy control group

UniProtKB/ Swiss-Prot	Symbol	Description	log_2 fold change			$–log_{10}P$-value		
			INSIP[*]	CVD-ILD[†]	Other ILD	INSIP	CVD-ILD	Other ILD
Q68D91	MBLAC2	Acyl-coenzyme A thioesterase MBLAC2	-2.18	-1.38	-2.64	4.57	3.77	4.49
Q00796	SORD	Sorbitol dehydrogenase	-4.73	-2.38	-5.16	4.54	1.75	4.95
P57737	CORO7	Coronin-7	-2.29	-1.10	-1.12	3.70	1.46	1.27
Q15389	ANGPT1	Angiopoietin-1	-4.80	-3.03	-5.05	3.64	1.71	4.06
A0A5B9<br>P01850	TRBC2;<br>TRBC1	T cell receptor beta constant 2<br>T cell receptor beta constant 1	-1.70	-1.28	-1.54	3.59	1.61	3.12
P06241	FYN	Tyrosine-protein kinase Fyn	-1.10	-1.67	-1.30	3.53	2.91	2.86
Q9NR46	SH3GLB2	Endophilin-B2	-2.45	-1.35	-2.92	3.43	1.94	3.76
Q9Y4D7	PLXND1	PLXND1	-1.94	-1.80	-1.98	3.29	2.53	2.69
P08709	F7	Coagulation factor VII	-2.10	-1.14	-1.69	3.28	2.73	3.54
Q15742	NAB2	NGFI-A-binding protein 2	-1.48	-1.28	-1.59	3.20	3.26	2.04
P05060	CHGB	Secretogranin-1	-2.39	-1.43	-1.97	2.99	3.24	3.32
Q8ND76	CCNY	Cyclin-Y	-2.92	-1.44	-2.18	2.97	1.92	2.51
Q7Z7A4	PXK	PX domain-containing protein kinase-like protein	-2.95	-2.90	-2.29	2.94	2.37	1.55
N/A	N/A	No corresponding protein name	-1.13	-1.26	-1.07	2.80	2.82	2.66
P02545	LMNA	Prelamin-A/C	-1.88	-1.09	-2.01	2.71	1.49	3.13
P41222	PTGDS	Prostaglandin-H2 D-isomerase	-1.62	-2.47	-2.32	2.58	4.32	2.37
P49419	ALDH7A1	Alpha-aminoadipic semialdehyde dehydrogenase	-2.58	-1.97	-1.89	2.56	2.11	1.56
Q9NY65	TUBA8	Tubulin alpha-8 chain	-1.19	-1.72	-1.99	2.52	3.45	2.87
Q92882	OSTF1	Osteoclast-stimulating factor 1	-1.33	-1.25	-1.16	2.50	2.56	2.75
P48061	CXCL12	Stromal cell-derived factor 1	-1.24	-1.52	-1.07	2.17	4.42	2.13
P16278	GLB1	Beta-galactosidase	-2.19	-2.07	-2.19	2.16	2.26	2.71
Q96IU4	ABHD14B	Putative protein-lysine deacylase ABHD14B	-1.50	-1.71	-2.61	2.14	2.18	2.96
P43403	ZAP70	Tyrosine-protein kinase ZAP-70	-2.27	-2.49	-3.37	2.06	1.93	3.40
Q96P63	SERPINB12	Serpin B12	-1.72	-1.16	-1.94	1.94	1.95	2.12
P01848	TRAC	T cell receptor alpha chain constant	-1.72	-2.36	-1.70	1.83	2.27	1.79
P46782	RPS5	40S ribosomal protein S5	-1.74	-2.01	-2.17	1.64	1.63	2.68
Q9UBQ7	GRHPR	Glyoxylate reductase/hydroxypyruvate reductase	-1.38	-3.46	-2.21	1.55	4.65	3.79
Q05397	PTK2	Focal adhesion kinase 1	-2.09	-2.96	-5.83	1.54	2.54	7.24
O60383	GDF9	Growth/differentiation factor 9	-2.00	-2.30	-1.71	1.43	1.56	1.22
Q14642	INPP5A	Inositol polyphosphate-5-phosphatase A	-1.29	-1.84	-1.58	1.30	1.67	2.22

[*]idiopathic nonspecific interstitial pneumonia; [†]collagen vascular disease-interstitial lung disease

Supplemental Table 5. *P* values in 2-group comparisons of SFTPB levels in serum EVs using Student's *t*-tests adjusted by Bonferroni correction

HC vs ILD								Non-PPF vs PPF			
All ILDs (Non-PPF)	All ILDs (PPF)	INSIP (Non-PPF)	INSIP (PPF)	CVD-ILD (Non-PPF)	CVD-ILD (PPF)	Other ILD (Non-PPF)	Other ILD (PPF)	All ILDs	INSIP	CVD-ILD	Other ILD
4.15×10^{-10}	1.37×10^{-13}	1.37×10^{-4}	9.06×10^{-7}	8.57×10^{-4}	8.34×10^{-21}	3.02×10^{-6}	7.26×10^{-7}	1.96×10^{-3}	6.65×10^{-1}	1.00×10^{-1}	5.63×10^{-2}

HC: healthy control, ILD: interstitial lung disease, PPF: progressive pulmonary fibrosis, INSIP: idiopathic non-specific interstitial pneumonia, CVD-ILD: collagen vascular disease-associated interstitial lung disease.

Supplemental Table 6. Patient characteristics for each ILD classification at the time of blood collection in the validation cohort

	INSIP* (non-PPF†) (n = 20)	INSIP (PPF) (n = 22)	CVD-ILD‡ (non-PPF) (n = 15)	CVD-ILD (PPF) (n = 14)	Other ILD (non-PPF) (n = 51)	Other ILD (PPF) (n = 20)
Age (years)	75 (7)	69 (10)	72 (12)	67 (15)	71 (10)	67 (11)
Sex						
Male	8 (40%)	13 (59%)	7 (47%)	9 (64%)	30 (59%)	12 (60%)
Female	12 (60%)	9 (41%)	8 (53%)	5 (36%)	21 (41%)	8 (40%)
Smoking history						
Current	0 (0%)	0 (0%)	0 (0%)	0 (0%)	0 (0%)	0 (0%)
Former	7 (35%)	10 (45%)	6 (40%)	8 (57%)	27 (53%)	12 (60%)
Never	11 (55%)	10 (45%)	8 (53%)	4 (29%)	18 (35%)	7 (35%)
N/A§	2 (10%)	2 (9%)	1 (7%)	2 (14%)	6 (12%)	1 (5%)
Classification of ILD						
INSIP	20 (100%)	22 (100%)	0 (0%)	0 (0%)	0 (0%)	0 (0%)
CVD-ILD	0 (0%)	0 (0%)	15 (100%)	14 (100%)	0 (0%)	0 (0%)
Unclassifiable ILD	0 (0%)	0 (0%)	0 (0%)	0 (0%)	36 (71%)	12 (60%)
FHP‖	0 (0%)	0 (0%)	0 (0%)	0 (0%)	0 (0%)	4 (20%)
PPFE**	0 (0%)	0 (0%)	0 (0%)	0 (0%)	3 (6%)	3 (15%)
Sarcoidosis	0 (0%)	0 (0%)	0 (0%)	0 (0%)	4 (8%)	1 (5%)
Others	0 (0%)	0 (0%)	0 (0%)	0 (0%)	8 (16%)	0 (0%)
Corticosteroid						
Yes	3 (15%)	3 (14%)	8 (53%)	8 (57%)	9 (18%)	3 (15%)
No	17 (85%)	19 (86%)	7 (47%)	6 (43%)	42 (82%)	17 (85%)
Immunosuppressant						
Yes	1 (5%)	0 (0%)	2 (13%)	4 (29%)	3 (6%)	2 (10%)
No	19 (95%)	22 (100%)	13 (87%)	10 (71%)	48 (94%)	18 (90%)
Antifibrotic therapy						
Yes	0 (0%)	1 (5%)	0 (0%)	1 (7%)	1 (2%)	3 (15%)
No	20 (100%)	21 (95%)	15 (100%)	13 (93%)	50 (98%)	17 (85%)
Radiological pattern of ILD						
UIP††	0 (0%)	0 (0%)	4 (27%)	2 (14%)	1 (2%)	2 (10%)
Probable UIP	0 (0%)	0 (0%)	1 (7%)	1 (7%)	0 (0%)	0 (0%)
Indeterminate UIP	0 (0%)	0 (0%)	0 (0%)	1 (7%)	19 (37%)	5 (25%)
Alternative diagnosis	20 (100%)	22 (100%)	10 (67%)	10 (71%)	31 (61%)	13 (65%)
NSIP	20 (100%)	22 (100%)	5 (33%)	10 (71%)	1 (2%)	1 (5%)
OP‡‡	0 (0%)	0 (0%)	0 (0%)	0 (0%)	3 (6%)	0 (0%)
HP	0 (0%)	0 (0%)	0 (0%)	0 (0%)	0 (0%)	4 (20%)
PPFE	0 (0%)	0 (0%)	0 (0%)	0 (0%)	3 (6%)	2 (10%)
Unclassifiable	0 (0%)	0 (0%)	5 (33%)	0 (0%)	24 (47%)	6 (30%)
Fibrosis region on CT§§						
< 10%	10 (50%)	9 (41%)	8 (53%)	2 (14%)	28 (55%)	3 (15%)
10–50%	9 (45%)	7 (32%)	7 (47%)	11 (79%)	19 (37%)	13 (65%)
> 50%	1 (5%)	6 (27%)	0 (0%)	1 (7%)	4 (8%)	4 (20%)
ILD-GAP‖‖ index						
0–1	16 (80%)	8 (36%)	9 (60%)	6 (43%)	2 (4%)	1 (5%)
2–3	1 (5%)	8 (36%)	3 (20%)	7 (50%)	21 (41%)	7 (35%)
4–5	0 (0%)	4 (18%)	0 (0%)	1 (7%)	6 (12%)	5 (25%)
> 5	0 (0%)	0 (0%)	0 (0%)	0 (0%)	1 (2%)	2 (10%)
N/A	3 (15%)	2 (9%)	3 (20%)	0 (0%)	21 (41%)	5 (25%)

Data are presented as the mean (standard deviation) or number of patients (percentage).
*idiopathic nonspecific interstitial pneumonia; †progressive pulmonary fibrosis; ‡collagen vascular disease-interstitial lung disease; §not available; ‖fibrotic hypersensitivity pneumonitis; **pleuroparenchymal fibroelastosis; ††usual interstitial pneumonia; ‡‡organizing pneumonia; §§computed tomography; ‖‖interstitial lung disease-gender age physiology

Supplemental Table 7. *P* values in 2-group comparisons of SFTPB levels in serum EVs by CT region analyzed using ANOVA and the Holm's method

HC vs <10%	HC vs 10–50%	HC vs >50%	<10% vs 10–50%	<10% vs >50%	10–50% vs >50%
1.30×10^{-10}	$<2.00 \times 10^{-16}$	$<2.00 \times 10^{-16}$	5.70×10^{-6}	5.70×10^{-6}	1.00×10^{-1}

HC: healthy control, ANOVA: analysis of variance

Supplemental Table 8. Validation of associations with non-IPF-ILD progression within 1 year by logistic regression model in the discovery cohort

	Univariate analysis by logistic regression model			Multivariate analysis by logistic regression model		
Variables	OR	95% CI	*P*	OR	95% CI	*P*
SFTPB (serum EVs)	1.69	1.02–2.79	0.042	1.41	0.83–2.39	0.20
KL-6 (serum)	1.00	1.00–1.00	0.60			
SP-D (serum)	1.00	1.00–1.00	0.38			
Age	0.99	0.94–1.03	0.53			
Sex	1.16	0.33–4.01	0.82			
Smoking history	0.53	0.16–1.79	0.31			
%FVC	0.96	0.93–0.99	0.0074	0.97	0.94–1.00	0.023
%DLco	0.98	0.95–1.00	0.072			

IPF: idiopathic pulmonary fibrosis, ILD: Interstitial lung diseases, OR: odds ratio, CI: confidence interval, EV: extracellular vesicle, FVC: forced vital capacity, DLco: diffusing capacity for carbon monoxide.

Supplemental Table 9. Characteristics of patients from whom lung tissue specimens were obtained for western blotting analysis

Case No.	Age (years)	Sex	Smoking history	Classification of ILDs	Resection area	Reason for resection
Control 1	68	F	Never	N/A	Right lower lobe	Surgery for lung cancer
Control 2	60	F	Never	N/A	Left upper lobe	Surgery for lung cancer
Control 3	69	F	Never	N/A	Right lower lobe	Surgery for lung cancer
Control 4	66	F	Never	N/A	Right lower lobe	Surgery for lung cancer
Control 5	68	M	Former	N/A	Left upper lobe	Surgery for lung cancer
PPF 1	71	M	Former	INSIP	Right lower lobe	Surgery for lung cancer
PPF 2	73	M	Former	Unclassifiable ILD	Right lower lobe	Surgery for lung cancer
PPF 3	42	F	Never	PPFE	Right upper lobe	Lung transplantation
PPF 4	62	M	Former	Unclassifiable ILD	Right upper lobe	Surgery for lung cancer
PPF 5	70	M	Former	Unclassifiable ILD	Right upper lobe	Surgery for lung cancer

ILD: interstitial lung disease, INSIP: idiopathic nonspecific interstitial pneumonia, N/A: not applicable, PPF: progressive pulmonary fibrosis, PPFE: pleuroparenchymal fibroelastosis

Supplemental Table 10. Characteristics of patients from whom serum and serum EV specimens were obtained for western blotting analysis

Case No.	Age (years)	Sex	Smoking history	Classification of ILDs
Control 1	32	M	Never	N/A (healthy volunteer)
Control 2	32	M	Never	N/A (healthy volunteer)
Control 3	34	F	Never	N/A (healthy volunteer)
Control 4	31	F	Never	N/A (healthy volunteer)
Control 5	33	F	Never	N/A (healthy volunteer)
PPF 1	70	M	Never	INSIP
PPF 2	67	M	Former	PPFE
PPF 3	70	F	Never	Unclassifiable ILD
PPF 4	60	F	Never	INSIP
PPF 5	71	F	Never	INSIP
PPF 6	76	F	Never	Sarcoidosis

ILD: interstitial lung disease, INSIP: idiopathic nonspecific interstitial pneumonia, N/A: not applicable, PPF: progressive pulmonary fibrosis, PPFE: pleuroparenchymal fibroelastosis

Supplemental Table 11. Primary antibodies used in this study

Antibodies	Source	Identifier
Anti-human pro-SFTPB	Santa Cruz Biotechnology	sc-133143
Anti-human Cpro-SFTPB	Santa Cruz Biotechnology	sc-133143
	Cloud Clone	PAB622Mu01
Anti-human mature SFTPB	Abcam	ab271345
Anti-human β-actin	Cell Signaling Technologies	#5125
Anti-mouse SFTPB	Cloud Clone	PAB622Mu01
Anti-mouse β-actin	Cell Signaling Technologies	#5125
HRP-conjugated anti–mouse IgG	GE Healthcare	NA931V
HRP-conjugated anti–rabbit IgG	GE Healthcare	NA934V

Supplemental Table 12

Cluster	Celltype	Lineage marker genes	References
0	Bcell	Cd19, Cd20	Carter RH., et al., Science, 1992
1	Endo_capillary	Cldn5 Pecam1	Gillich A., et al., Nature, 2020
2	Bcell	Cd19, Cd20	Carter RH., et al., Science, 1992
3	Endo_capillary	Cldn5 Pecam1	Gillich A., et al., Nature, 2020
4	Endo_capillary	Cldn5 Pecam1	Gillich A., et al., Nature, 2020
5	Tcell_CD4T	Cd3e Cd4 Trbc1	Szabo PA. et al., Nat Commun., 2019
6	MoMac	Cd14 Csf1r Itgam	Yona S., et al., Immunity 2013
7	Tcell_CD8T	Cd3e Cd8 Trbc1	Szabo PA. et al., Nat Commun., 2019
8	NK	Tbx21 Klrb1c, Ncr1	Grégoire C., et al Immunol Rev 2007
9	Epi_AT2	Sftpc Epcam	Perl AK., et al., Proc. Natl. Acad. Sci. 2002
10	Fibroblast	Col1a1 Pdgfra	Liu, X., et al., BioRxiv, 2020
11	Bcell	Cd19, Cd20	Carter RH., et al., Science, 1992
12	Fibroblast	Col1a1 Pdgfra	Liu, X., et al., BioRxiv, 2020
13	MoMac	Cd14 Csf1r Itgam	Yona S., et al., Immunity 2013
14	Neutrophil	S100a8 S100a9 Csf3r	Zilionis R., et al, Immunity 2019
15	Fibroblast	Col1a1 Pdgfra	Liu, X., et al., BioRxiv, 2020
16	MoMac	Cd14 Csf1r Itgam	Yona S., et al., Immunity 2013
17	Tcell_CD4T	Cd3e Cd4 Trbc1	Szabo PA. et al., Nat Commun., 2019
18	MoMac	Cd14 Csf1r Itgam	Yona S., et al., Immunity 2013
19	Endo_aerocyte	Pecam1 Car4 Cldn5	Gillich A., et al., Nature, 2020
20	Fibroblast	Col1a1 Pdgfra	Liu, X., et al., BioRxiv, 2020
21	DC_cDC2	Cd209a Itgax	Schlitzer A, et al., Nat Immunol 2015
22	Bcell	Cd19, Cd20	Carter RH., et al., Science, 1992
23	MoMac	Cd14 Csf1r Itgam	Yona S., et al., Immunity 2013
24	Epi_AT1	Ager Hopx Epcam	Flodby P., et al. Am. J. Respir. Cell Mol. Biol. 2010, Barkauskas CE., et al. J. Clin. Invest., 2013.
25	MoMac	Cd14 Csf1r Itgam	Yona S., et al., Immunity 2013

26	SMC	Acta2 Myh11	Wakasugi T., et al., PLoS One, 2019
27	Tcell_Treg	Foxp3 Cd3e Cd4	Painter MW et al., J Immunol, 2011
28	Pericyte	Notch3 Pdgfrb	Kato K., et al., Nat Commun., 2018
29	Tcell_Tgd	Trdc Trgc1 Cd3e	Kranz D. M., et al Nature 1985
30	Epi_ciliated_clara	Scgb1a1 Foxj1	Evans MJ., et al. Lab Invest., 1978, Blatt EN., et al., Am J Respir Cell Mol Biol, 1999.
31	MoMac	Cd14 Csf1r Itgam	Yona S., et al., Immunity 2013
32	Epi_AT1	Ager Hopx Epcam	Flodby P., et al. Am. J. Respir. Cell Mol. Biol. 2010, Barkauskas CE., et al. J. Clin. Invest., 2013.
33	ILC2	Il1rl1	Moro K., et al., Nat Protocol 2015
34	Fibroblast	Col1a1 Pdgfra	Liu, X., et al., BioRxiv, 2020
35	Endo_LEC	Prox1	Wigle JT and Oliver G, Cell, 1999
36	Endo_venous	Pecam1 Vwf, Bst1 (CD1	Goveia J., et al., Cancer Cell 2020, Wakabayashi T, et al Cell Stem Cell 2018
37	Mac_DC proliferate	Mki67 Itgam	
38	Fibroblast	Col1a1 Pdgfra	Liu, X., et al., BioRxiv, 2020
39	Tcell_Tgd	Trdc Trgc1 Cd3e	Kranz D. M., et al Nature 1985
40	DC_cDC1	Xcr1 Itgae	Schlitzer A, et al., Nat Immunol 2015
41	Epi_ciliated_clara	Scgb1a1 Foxj1	Evans MJ., et al. Lab Invest., 1978, Blatt EN., et al., Am J Respir Cell Mol Biol, 1999.
42	MoMac	Cd14 Csf1r Itgam	Yona S., et al., Immunity 2013
43	doublet		
44	doublet		
45	Neutrophil	S100a8 S100a9 Csf3r	Zilionis R., et al, Immunity 2019
46	Bcell	Cd19, Cd20	Carter RH., et al., Science, 1992
47	doublet		
48	Endo_arterial	Gja5 Bmx Pecam1	Gillich A., et al., Nature 2020
49	Fibroblast	Col1a1 Pdgfra	Liu, X., et al., BioRxiv, 2020
50	DC_cDC3	Fscn1 Ccr7	Schlitzer A, et al., Nat Immunol 2015
51	Tcell_CD8T	Cd3e Cd8 Trbc1	Szabo PA. et al., Nat Commun., 2019
52	Fibroblast_Peribronchiolar	Hhip Acta2	Tsukui T. et al Nat Commune 2020
53	DC_pDC	Siglech	Schlitzer A, et al., Nat Immunol 2015
54	Tcell_Tgd	Trdc Trgc1 Cd3e	Kranz D. M., et al Nature 1985

55	Tcell_proliferated	Cd3e Mki67	
56	Eosinophil	Siglecf	
57	NK	Tbx21 Klrb1c, Ncr1	Grégoire C., et al Immunol Rev 2007
58	Fibroblast	Col1a1 Pdgfra	Liu, X., et al., BioRxiv, 2020
59	Platelets	Hbb-b1	
60	misc		
61	Epi_ciliated_clara	Scgb1a1 Foxj1	Evans MJ., et al. Lab Invest., 1978, Blatt EN., et al., Am J Respir Cell Mol Biol, 1999.
62	Bcell	Cd19, Cd20	Carter RH., et al., Science, 1992
63	Mesothelial	Msln Wt1	Dixit R., et al. Development 2013
64	doublet		
65	doublet		
66	Basophil	Fcer1a Mcpt8	Sullivan BM., et al. Nat Immunol 2011
67	Plasma cell	Ighm	
68	Bcell	Cd19, Cd20	Carter RH., et al., Science, 1992
69	doublet		
70	Fibroblast	Col1a1 Pdgfra	Liu, X., et al., BioRxiv, 2020
71	NK	Tbx21 Klrb1c, Ncr1	Grégoire C., et al Immunol Rev 2007
72	Fibroblast	Col1a1 Pdgfra	Liu, X., et al., BioRxiv, 2020
73	Bcell	Cd19, Cd20	Carter RH., et al., Science, 1992
74	Tcell_CD8T	Cd3e Cd8 Trbc1	Szabo PA. et al., Nat Commun., 2019
75	Endo_proliferated	Pecam1 Mki67	
76	Pericyte	Notch3 Pdgfrb	Kato K., et al., Nat Commun., 2018
77	Neutrophil	S100a8 S100a9 Csf3r	Zilionis R., et al, Immunity 2019
78	Tcell_CD4T	Cd3e Cd4 Trbc1	Szabo PA. et al., Nat Commun., 2019
79	Sox10pos_cells	Sox10	
80	Epi_AT2	Sftpc Epcam	Perl AK., et al., Proc. Natl. Acad. Sci. 2002
81	Pro-platelet	Ppbp	
82	doublet		
