# Supplementary material for: SFTPB in serum extracellular vesicles as a biomarker of progressive pulmonary fibrosis
Source: JCI Insight. 2024 Jun 10;9(11):e177937. doi: 10.1172/jci.insight.177937 (PMC11382876; doi:10.1172/jci.insight.177937)
Supplement: Unedited blot and gel images [file jciinsight-9-177937-s166.pdf]

Full unedited gel for Figure 5B

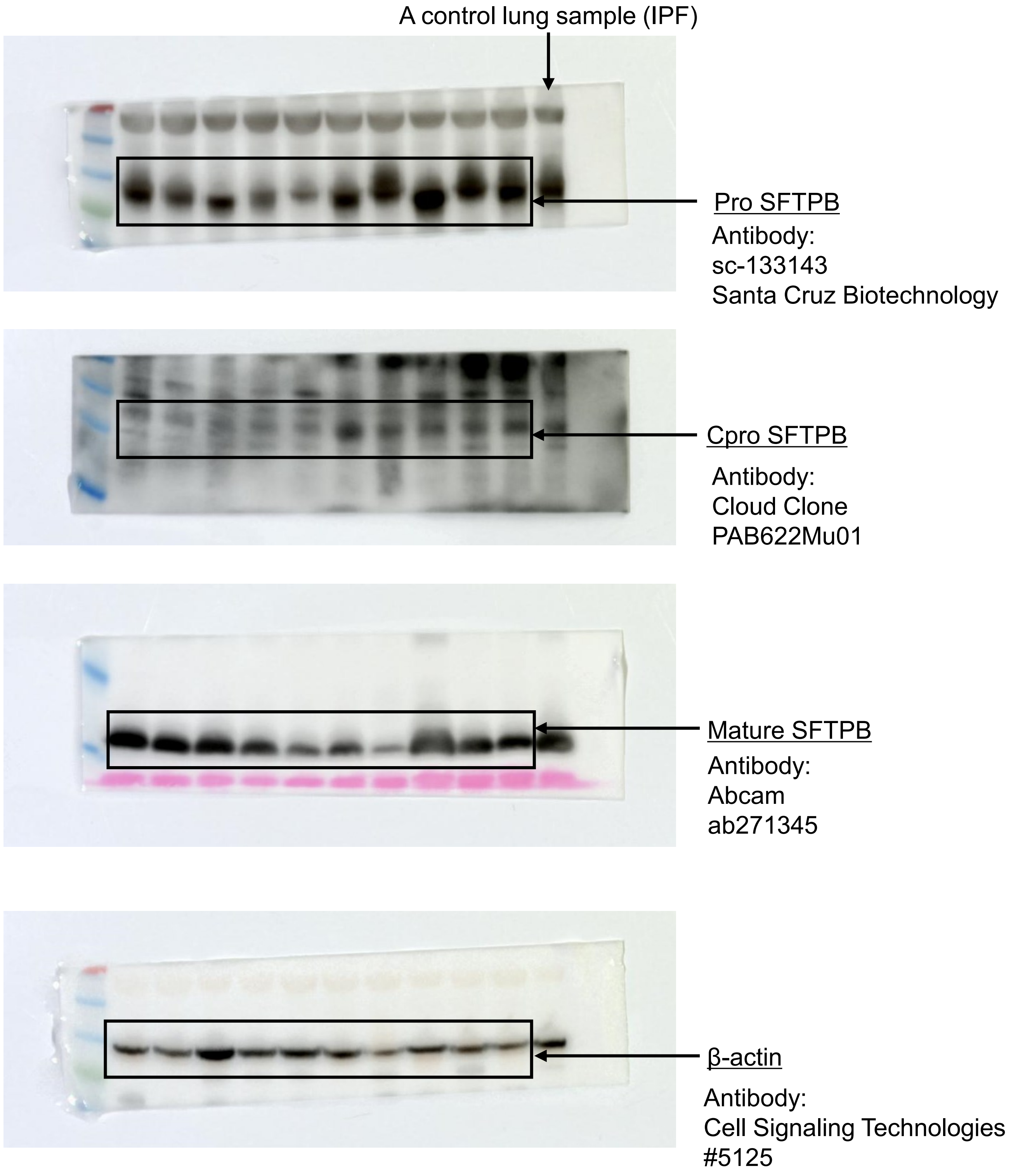

Full unedited gel for Figure 5F

A control lung sample

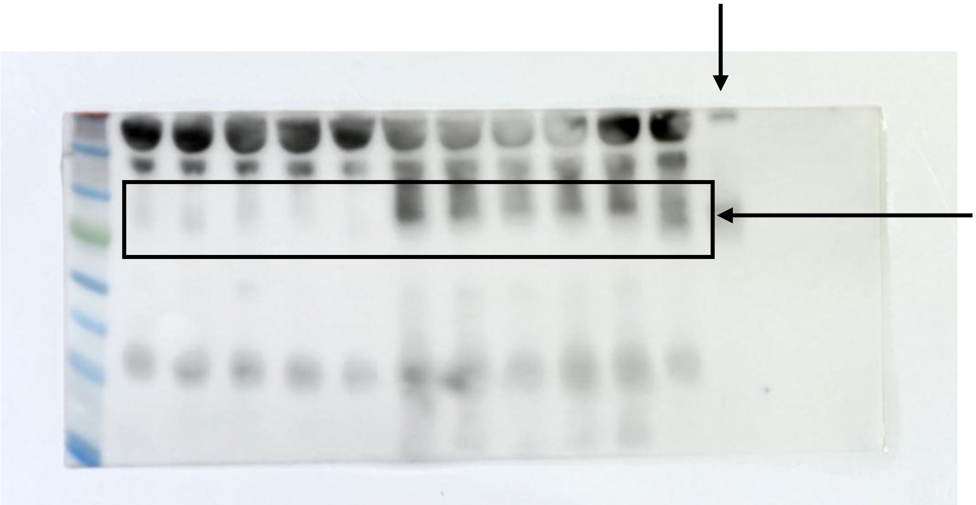

Pro SFTPB

Antibody:
sc-133143
Santa Cruz Biotechnology

A control lung sample

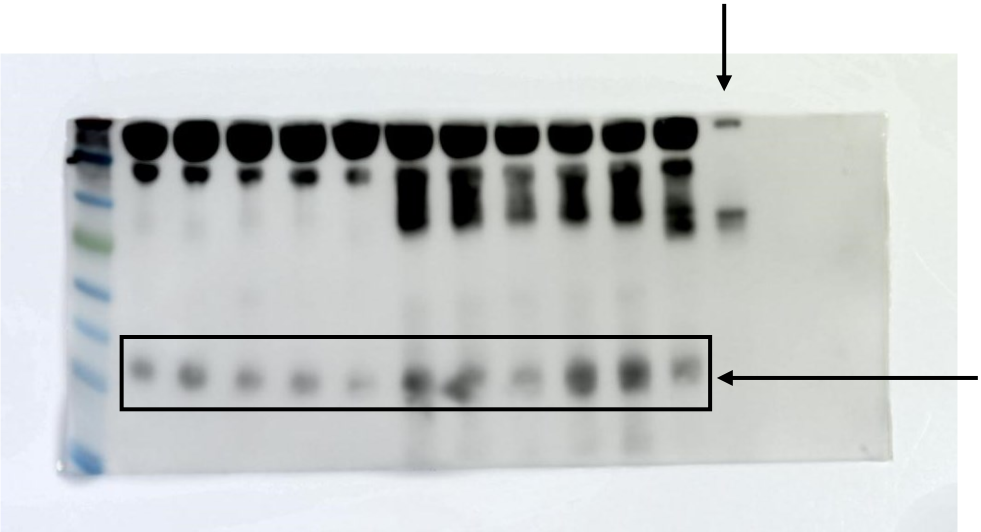

Cpro SFTPB

Antibody:
sc-133143
Santa Cruz Biotechnology

Full unedited gel for Figure 5I

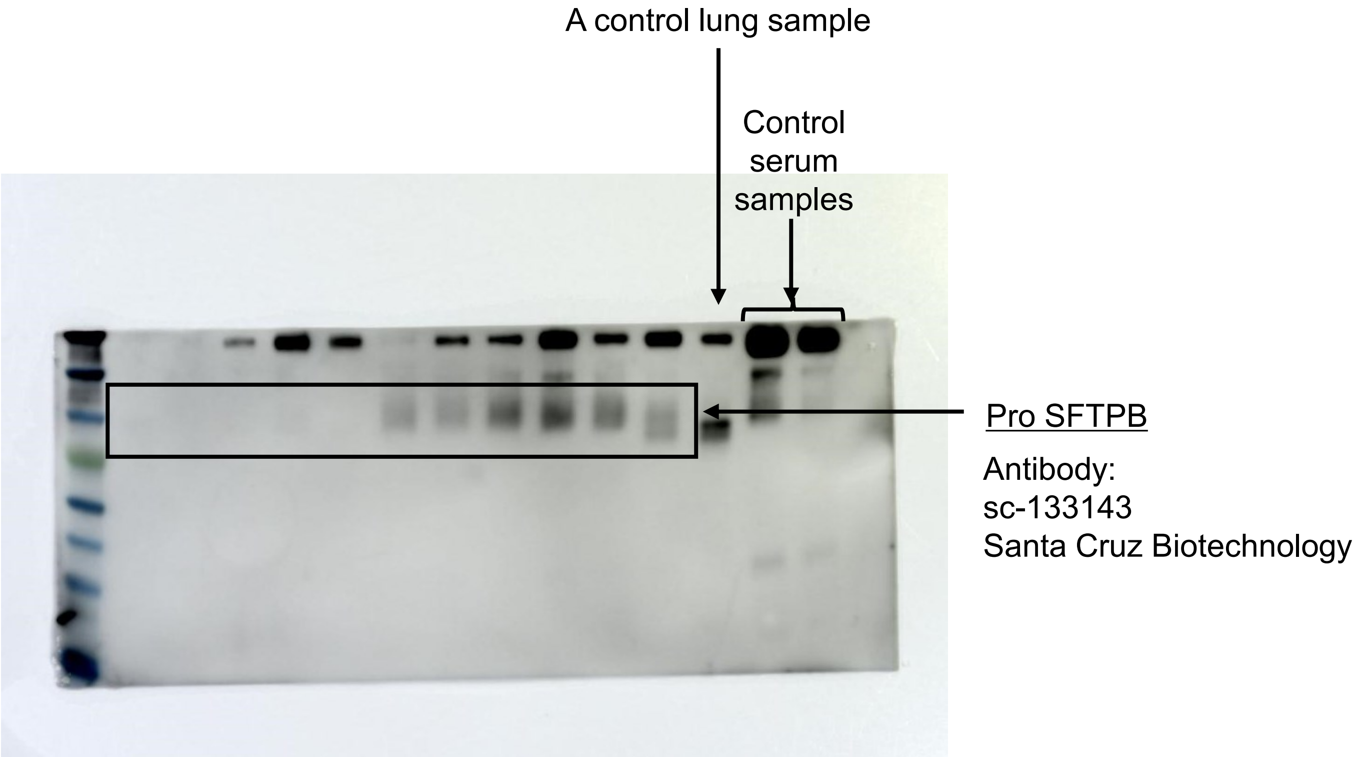

Full unedited gel for Figure 5K

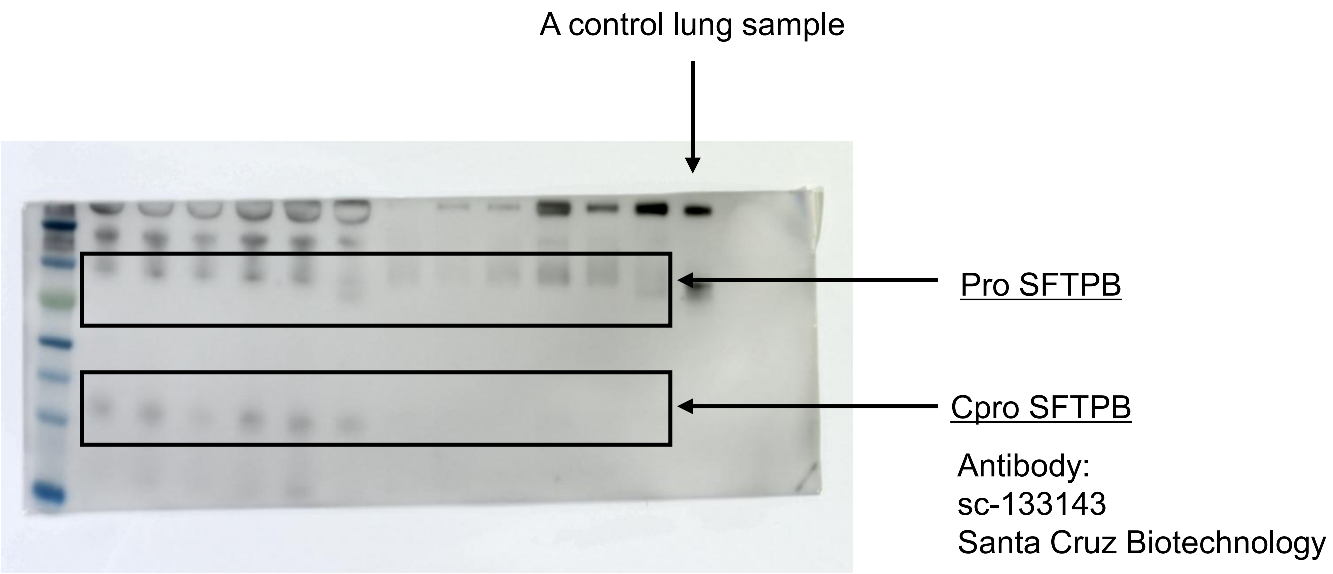

Full unedited gel for Figure 6

B

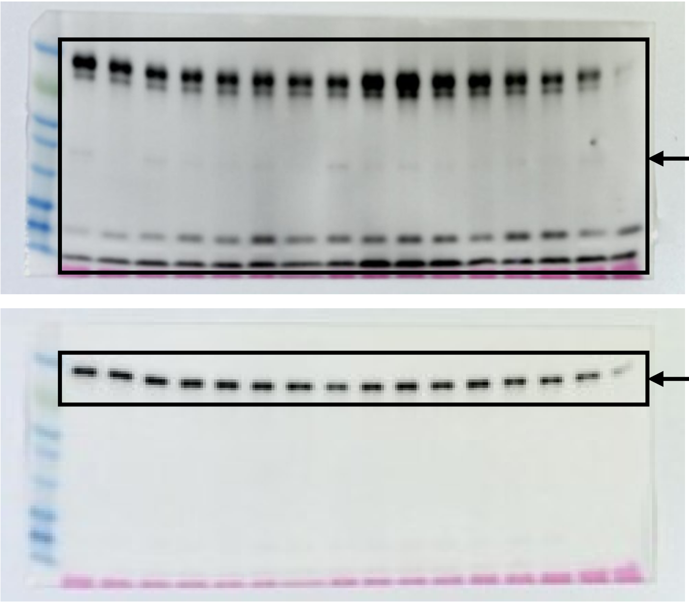

SFTPB
Antibody:
Cloud Clone
PAB622Mu01

β-actin
Antibody:
Cell Signaling Technologies
#5125

C

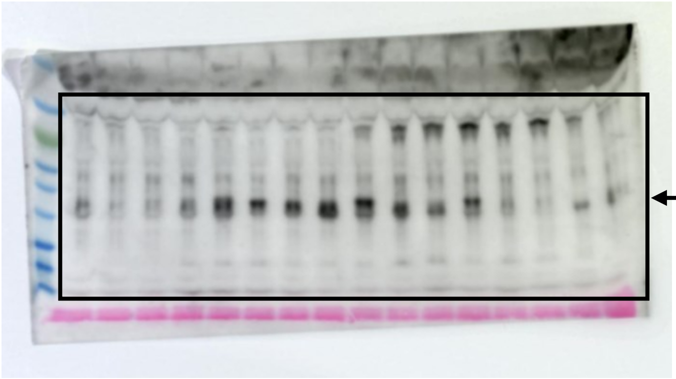

Antibody:
Cloud Clone
PAB622Mu01

D

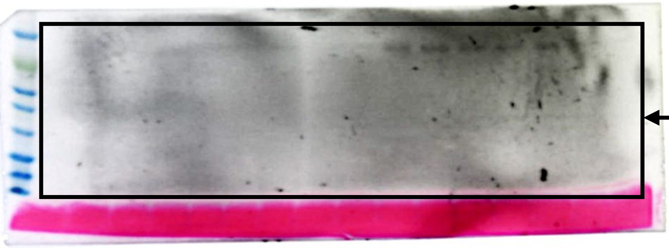

Antibody:
Cloud Clone
PAB622Mu01

E

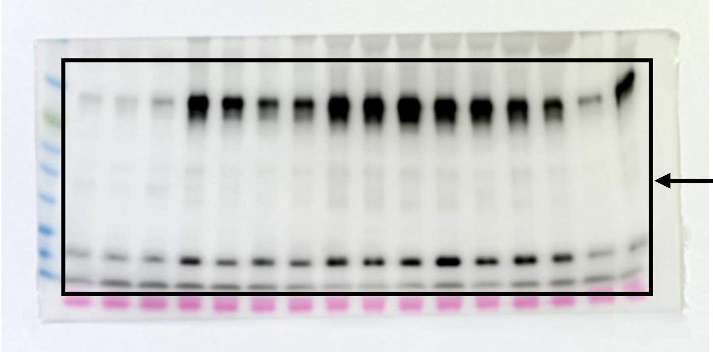

Antibody:
Cloud Clone
PAB622Mu01

Full unedited gel for Supplemental Figure 2A

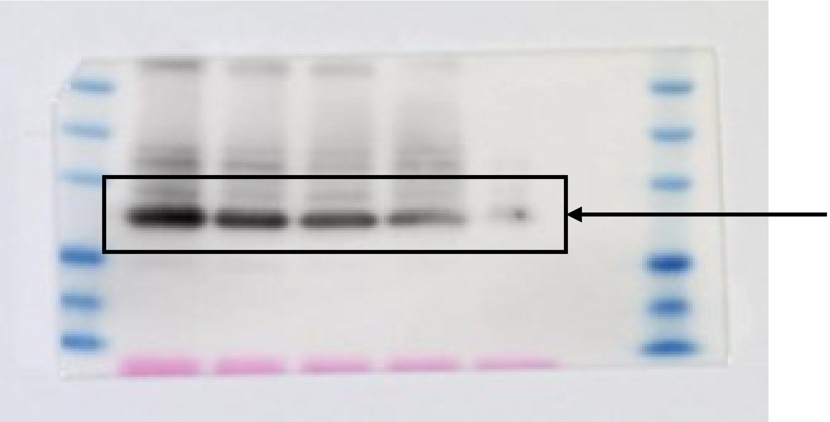

CD9
Antibody:
Invitrogen
AHS0902

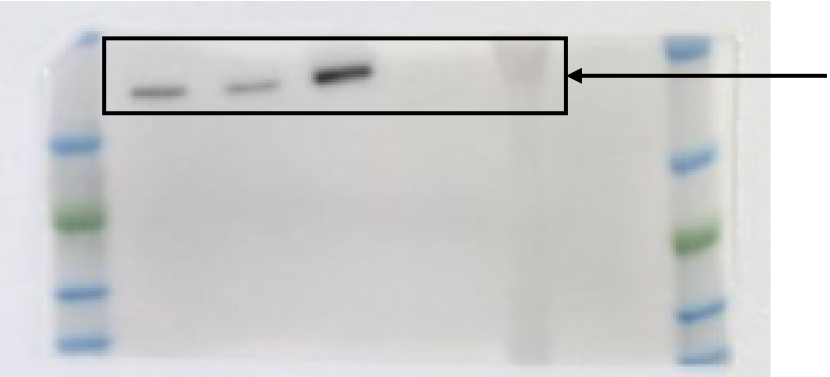

Flotillin
Antibody:
Proteintech
15571-1-AP

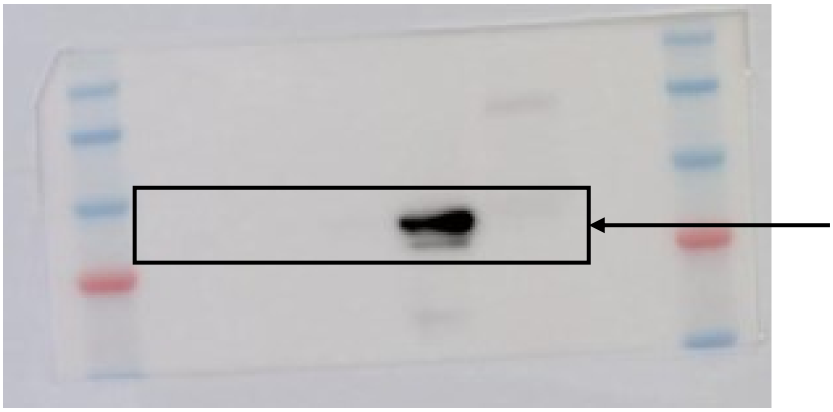

Calnexin
Antibody:
Abcam
ab22595

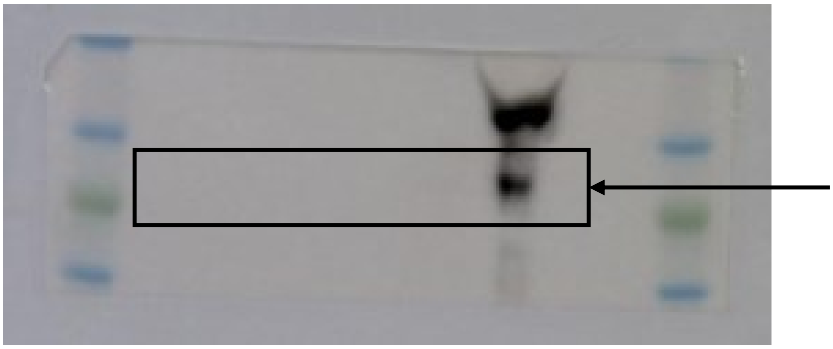

Haptoglobin
Antibody:
Abcam
ab131236
